# Affordable Prices Without Threatening the Oncological R&D Pipeline—An Economic Experiment on Transparency in Price Negotiations

**DOI:** 10.1158/2767-9764.CRC-21-0031

**Published:** 2022-01-27

**Authors:** Nora Franzen, Andreas Ziegler, Giorgia Romagnoli, Valesca P. Retèl, Theo J.S. Offerman, Wim H. van Harten

**Affiliations:** 1 The Netherlands Cancer Institute, Department of Psychosocial Research and Epidemiology, Amsterdam, The Netherlands.; 2 University of Twente, Department of Health Technology and Services Research, Enschede, The Netherlands.; 3 Center for Research in Experimental Economics and Political Decision Making, University of Amsterdam, Amsterdam, the Netherlands.; 4 Rijnstate Hospital, Arnhem, the Netherlands.

## Abstract

The high prices of innovative medicines endanger access to care worldwide. Sustainable prices need to be affordable while sufficiently incentivizing research and development (R&D) investments. A proposed solution is increased transparency. Proponents argue that price and R&D cost confidentiality are drivers of high prices. On the contrary, supporters of confidentiality claim that confidentiality enables targeted discounts which make treatments affordable; moreover, pharmaceutical companies argue that R&D investments would suffer with more transparency.

Despite the political relevance, limited empirical evidence exists on the effects of transparency regulations. We contribute to fill this gap with an experiment where we replicate the EU pharmaceutical market in a laboratory setting. In a randomized controlled study, we analyzed how participants, 400 students located in four European countries, negotiated in the current system of *Price Secrecy* in comparison with innovative bargaining settings where either prices only *(Price Transparency)* or prices and R&D costs *(Full Transparency)* were made transparent to buyers. We found that *Price transparency* had no statistically significant effect on average prices or number of patients treated and made R&D investments significantly smaller (−16.86%; *P*: 0.0024). On the other hand, *Full Transparency* reduced prices (−26%; *P*: 0.0004) and held the number of patients constant at the level of *Price Secrecy*. It produced price convergence between countries with low and high health budgets, and, despite lower prices, had no effect on R&D investments.

Our findings provide novel evidence that combining price and R&D cost transparency could be an effective policy to contribute to sustainable medicine prices.

See related article by Franzen et al. (Cancer Discov 2022;12:299–302).

## Introduction

Affordable medical treatments are key for widespread health, well-being, and solidarity within and across nations. Excessive prices of anticancer treatments and their impact on the affordability, availability, and accessibility of medicines have been high on the political agenda at the national and international level (1, 2).

The pharmaceutical market is characterized by conspicuous informational asymmetries, with pharmaceutical companies having a more precise assessment of key factors such as actual prices (3), research and development (R&D) costs (4), and clinical trial data (5, 6). In the present system, the outcomes of bilateral negotiations remain confidential. Buyers do not know how much payers in neighboring countries or even in the same health care system spend on a particular medicine. While accessible official price lists exist, confidential price discounts of list prices are significant, most commonly higher than 20%, and range widely (3). Moreover, prices seem to be correlated with, but not clearly follow wealth distributions across countries (7). The costs of R&D are also confidential, and there is considerable imprecision in their failure adjusted estimates per newly developed medicine, ranging from USD 648 (4) to USD 2870 mn (8) and even higher in oncology.

Increased transparency is one of the solutions proposed to reduce prices (9). Its proponents claim that it would improve the overall fairness and efficiency of the current price negotiation system (10, 11). Currently, prices differ between health care providers and are set with no transparent relation with development, production, and distribution costs, nor with the purchasing power of different countries. The policy push toward transparency is taking place both at the EU level and globally. In the United States, transparency legislation was implemented to protect patients from the consequences of financial hardship resulting from overly expensive pharmaceutical treatments (12). Transparency of R&D costs is mentioned as an objective of the EU pharmaceutical strategy (13). In 2019, the World Health Assembly approved a milestone resolution on pharmaceutical transparency (14), but countries have been slow to install respective measures. It is argued that transparency could increase the negotiation power of payers in monopolies and oligopolies (15).

On the other hand, supporters of confidentiality claim that price confidentiality allows for targeted discounts and therefore improves the affordability of medicines (16). The theoretical argument is that transparency may prevent differential pricing and converging prices may not be affordable for low- and middle-income countries. Even stakeholders in higher-health-budget countries have expressed concerns that transparent prices may prevent them from reaching perceived favorable prices if discounts become transparent (17). On top of this, the pharmaceutical industry warns policy makers that transparency of R&D costs, to the extent that it leads to lower prices, may also discourage investment in R&D (16).

Despite the extreme relevance of this debate, the policy discussion has primarily centered on these theoretical and counterfactual arguments of opponents and proponents of drug price transparency. Little empirical evidence exists that shows the effect of transparency legislations of medicine prices and the current secretive system in itself largely prevents a precise factual analysis of medicine prices (18). From a theoretical viewpoint, the conversation also appears inconclusive. Bargaining is a central area in the field of economics, but existing models are silent about bargaining outcomes in the specific institutional context under consideration. Moreover, a purely theoretical economic analysis, based on classical assumptions such as selfish motives and individual rationality, would also be silent or, at best, speculative about the interaction of transparency with the acceptability of price discounts to low-income and middle-income countries. When considering the viability of transparent price differences between countries, psychologic factors and value judgments about what constitutes fair pricing are likely to play an important role. These considerations invite an empirical investigation.

This study's objective is to empirically test the effects of price and R&D cost transparency on prices and R&D investments in a European setting. We do so with an economic experiment. While adopting a game theoretic framework is increasingly used to help decision making in medicine [see, e.g., Archetti and colleagues (19), McFadden and colleagues (20), and Stanková and colleagues (21)], it has, to our knowledge, not been applied to evaluate price and R&D cost transparency policies in markets akin to the pharmaceutical industry.

## Materials and Methods

We run an economic experiment using informed cohorts of established behavioral economic laboratories in a cross-country study involving participants from the Netherlands, Germany, Poland, and Spain. The experiment is based on a replication of the pharmaceutical market as an economic bargaining game (22). We adopted the standard game theoretic framework in which players’ decisions and interactions are mapped into predefined and known consequences. This experiment and its analysis were preregistered at the American Economic Associations’ RCT registry for experiments, with ID AEARCTR-0007230 (https://doi.org/10.1257/rct.7230–1.0).

Experimental negotiations take place within the well-established design of double auction markets, which are widely used in experimental economics. Originally established and tested by Nobel prize winner Vernon Smith, these market institutions have repeatedly shown to converge to perfectly competitive equilibrium outcomes (23, 24). Within this framework, we contribute by modeling essential features of the pharmaceutical market suitable to study the role of transparency. These features have been carefully selected in the preparatory phase with experts in drug price negotiations, cancer research, and health economics.

### Design

In a randomized controlled laboratory experiment, we compared a control arm (A1: *Price Secrecy*), with two alternative institutional scenarios characterized by progressively larger degrees of transparency. In the second experimental arm (A2: *Price Transparency*), prices concluded by other countries were made transparent to all market participants; in the third experimental arm *(*A3: *Full Transparency*), both prices and (verifiable) R&D costs were made transparent.

Experimental groups comprise five participants: four buying countries (Germany, the Netherlands, Spain, Poland) involved in repeated negotiations with one pharmaceutical company over the price of an “innovative and highly effective anticancer medicine.” Within this game, we capture essential features of the pharmaceutical market such as asymmetric information, different financial capacities of buying countries, repeated investment decisions of pharmaceutical companies as well as repeated interaction with countries in price negotiations.

### Participants, Incentives, and Experimental Parameters

Experimental participants were students recruited to take the role of a pharmaceutical company or one of the four buying countries. The usage of student population is a well-established method in economics and treatment effects found with students usually carry over to studies that use professionals (25, 26). Samples of appropriate size to ensure well-powered studies are widely available, well-established best practices exist, and the student samples have repeatedly proven to understand scenarios in similar ways as professionals do (27, 28). Also, behaviors of student samples are generally in line with behaviors of representative samples (29). To support the external validity of our findings, it was deemed important to capture cultural dynamics and realistic degrees of solidarity across countries (30). Hence, country representatives in negotiations were recruited from laboratories located in the respective countries. Participants taking the role of the pharmaceutical industry were recruited from an international pool of subjects of a laboratory in Germany. To encourage participants to take the decisions seriously, they earned real-world money depending on their performance in the experiment. Moreover, decisions taken in the experiment were given real-world consequences on access to medical treatment broadly defined. As we later explain, these took the form of donations towards cancer research institutes located in the respective countries.

We have modeled the market structure and incentives of the real-world markets by inducing laboratory values consistent with those found in real pharmaceutical market (31). Countries differed in population size, available budget, and maximal willingness to pay (WTP) for each patient. Experimental parameters were set to match real-world values. In Table 1, we show the parameters utilized (expressed in experimental points). In the exposition, we refer to Poland and Spain as low-health-budget countries and Germany and the Netherlands as higher-health-budget countries.

**TABLE 1:** Experimental parameters (WTP and budget in points).

Country	Health budget level	WTP per patient	# patients	Budget
The Netherlands	High	80	13	650
Germany	High	90	20	1,100
Spain	Low	30	19	340
Poland	Low	25	18	300

### Timeline of the Experiment

The experiment starts with a learning period. The main analysis is then based on 10 repetitions (i.e., *periods*) of the following sequence of stages:

- Stage 1: The pharmaceutical company decides the maximal amount of money to invest in R&D and develop a medicine conditional on the size of the R&D costs that vary from period to period.
- Stage 2: Conditional on the development of the medicine, several communication channels open where players can verbally discuss nonbinding price agreements among themselves.
- Stage 3: A formal bilateral bargaining stage opens where the pharmaceutical company and each country simultaneously submit offers and counteroffers and agreement is binding. Players have 4 minutes to reach an agreement, after which negotiations fail.

If at stage 1 the pharmaceutical company decides not to develop the medicine, a communication channel opens among players, after which the experiment moves to the next period. See Supplementary Data S1 for more details.

### Experimental Arms and Information Available to Participants

Participants were randomly allocated to one among three experimental arms. All arms followed the same structure and only differed in the amount of information observed or exchangeable during the experiment:

- **Experimental arm 1** – control group *“Price Secrecy”*: Information on R&D costs was confidential and only available for participants in the role of the pharmaceutical company. Information about negotiated and concluded prices was not revealed to other countries at any point of the study. Countries were also not allowed to share this information with other countries.
- **Experimental arm 2** – intervention *“Price Transparency”*: Price offers and counteroffers of all participants were instantaneously revealed during the negotiations, and a summary of all agreed prices was provided at the end of each period. Countries were allowed to share price information. Information on R&D costs remained confidential and was only available for participants in the role of the pharmaceutical company.
- **Experimental arm 3** – intervention “*Full Transparency”*: In addition to price transparency implemented as in experimental arm 2, the R&D costs paid in every period were truthfully communicated to all countries before the negotiations took place; moreover, participants’ payoffs were revealed to all at the end of each period.

### Main Outcome Measures, Data Analysis, and Experimental Design

The main outcomes of interest, focal elements of the policy debate, are agreed price levels (mean) and R&D investment levels (mean maximal willingness to invest in R&D). We further study price dispersion (variance) to explore whether more transparency leads to price convergence across countries. Finally, we map these outcomes into economic measures of individual and aggregate welfare, both of which are key drivers of policy considerations.

Aggregate welfare is the main measure of market efficiency because distributional effects can theoretically be offset with ex-post redistributions via taxes and subsidies. When the political viability of ex-post redistribution is questioned, it is also important to evaluate individual welfare effects. Thus, we also highlight the distributional effects of the different experimental arms.

Individual welfare for the countries is calculated as the sum, over all treated patients, of the WTP for each patient (interpreted as the value assigned by the country to the medical treatment of its citizens) minus the price of the treatment negotiated with the pharmaceutical company. Individual welfare for the pharmaceutical company is measured by the company's profits, so it is given by revenues (the number of treatments sold multiplied by the price of each treatment) minus costs (R&D costs plus production costs). Individual welfare coincides with the monetary payoffs earned by the players, and is formally described in Supplementary Data S1.

Aggregate welfare is computed as the sum of individual welfare over all participants. This approach corresponds to the standard method of measuring welfare from an economic perspective. A complementary measure of aggregate welfare—relevant for the medical application we study—is the overall number of patients treated. This approach abstracts away from differences in WTP across countries and thus treats each patient as having equal weight.

In terms of analysis, nonparametric tests (Mann–Whitney *U* tests) were used to compare outcomes between experimental arms. To check the robustness of the nonparametric tests, we run regressions of the outcome variables on treatment dummies, clustering SEs at the group level. As controls, we add survey-elicited (preference) measures on participants willingness to take risks, their altruism, competitiveness as well as gender, age, and study type (32, 33). In addition, we analyzed how prices respond to R&D costs Ordinary Least Squares regression (OLS).

Across all experimental arms, price agreement was almost always reached (in 98.4% of cases or more). Therefore, we only analyze prices for periods and countries where agreement was reached. The experiment and its analysis were preregistered at the American Economic Associations’ RCT registry for experiments and a detailed analysis plan can be found there (34). The set-up of the market game, the experimental timeline, the main outcomes, and the experimental implementation can be found in the Supplementary Data S1, the instructions to the participants in the Supplementary Data S2. Prior to running, the program was tested with Dutch and international participants. There were no real patients involved and data were anonymized. Institutional Review Board approval was obtained from the Economics & Business Ethics Committee at the University of Amsterdam (Amsterdam, the Netherlands; reference EC 20210201060246, February 1, 2021).

### Data Availability Statement

The raw data generated in this study are publicly available in Zenodo at https://doi.org/10.5281/zenodo.5795132. A clean version will be published in February 2022 in the same repository.

## Results

A total of 400 students passed the prerequirements of the experiment and completed the experiment. The share of participants not passing the required comprehension quiz was 1.5%. There was no difference in the experimental arms in terms of educational status, gender, age, willingness to take risks, altruism, or competitiveness (when regressing each characteristic on treatment dummies, no coefficient on the dummies are significant at a *P* < 0.1; for educational status, we use Pearson *χ*^2^ test). Here, we present a summary of the main findings. For extended tables, please refer to the Supplementary Data S3. Results are presented in terms of percentage differences between the control arm *Price Secrecy* (A1) and the two intervention arms *Price Transparency* (A2), *and Full Transparency* (A3).

### Effects on Prices

#### Mean Prices

**FIGURE 1:**
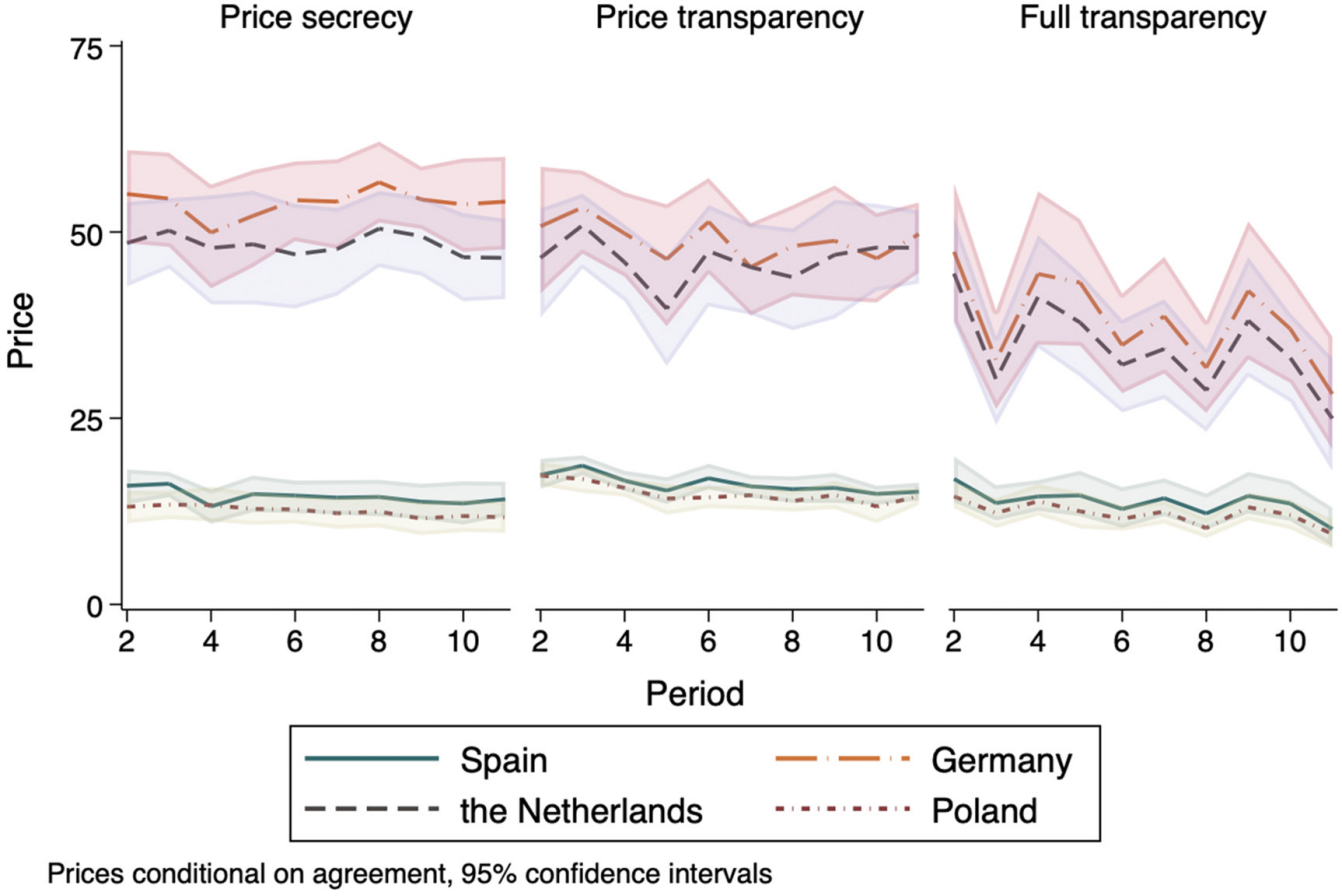
Average prices per arm, points per period. Notes: Lines represent average prices per period, per country, for each arm. The shaded areas are 95% bootstrapped confidence intervals.

In *Price Transparency,* no statistically significant difference in overall average prices was found (A2: −0.03%; *P*: 0.640), with some indication of price increases for countries in the lower-health-budget group (A2: +15.78%; *P*: 0.051). In *Full Transparency,* average prices decreased significantly for all countries (A3: −26%; *P* < 0.001), with stronger reductions for countries in the higher-health-budget group (A3: −31%; *P* < 0.001) and not statistically different prices for lower-health-budget countries compared with the control arm. Figure 1 presents the evolution of average prices per arm over the 10 periods of the experiment.

#### Price Convergence

Evidence for some price convergence is found both with *Price Transparency* and *Full Transparency* as compared with *Price Secrecy.* For *Price Transparency*, the variance of prices decreases by 26.33% (*P*: 0.012), and for *Full Transparency* by 55.49% (*P* < 0.001). In addition, in *Price Transparency* we observe additional convergence within the group of lower-health-budget countries (*P*: 0.017). Conversely, in *Full Transparency* the variance of lower-health-budget countries is similar to *Price Secrecy* (*P*: 0.200), while within-group convergence is found for higher-health-budget countries (*P*: 0.009).

### Effects on Investments in R&D and on the Relationship Between R&D Costs and Prices

**FIGURE 2:**
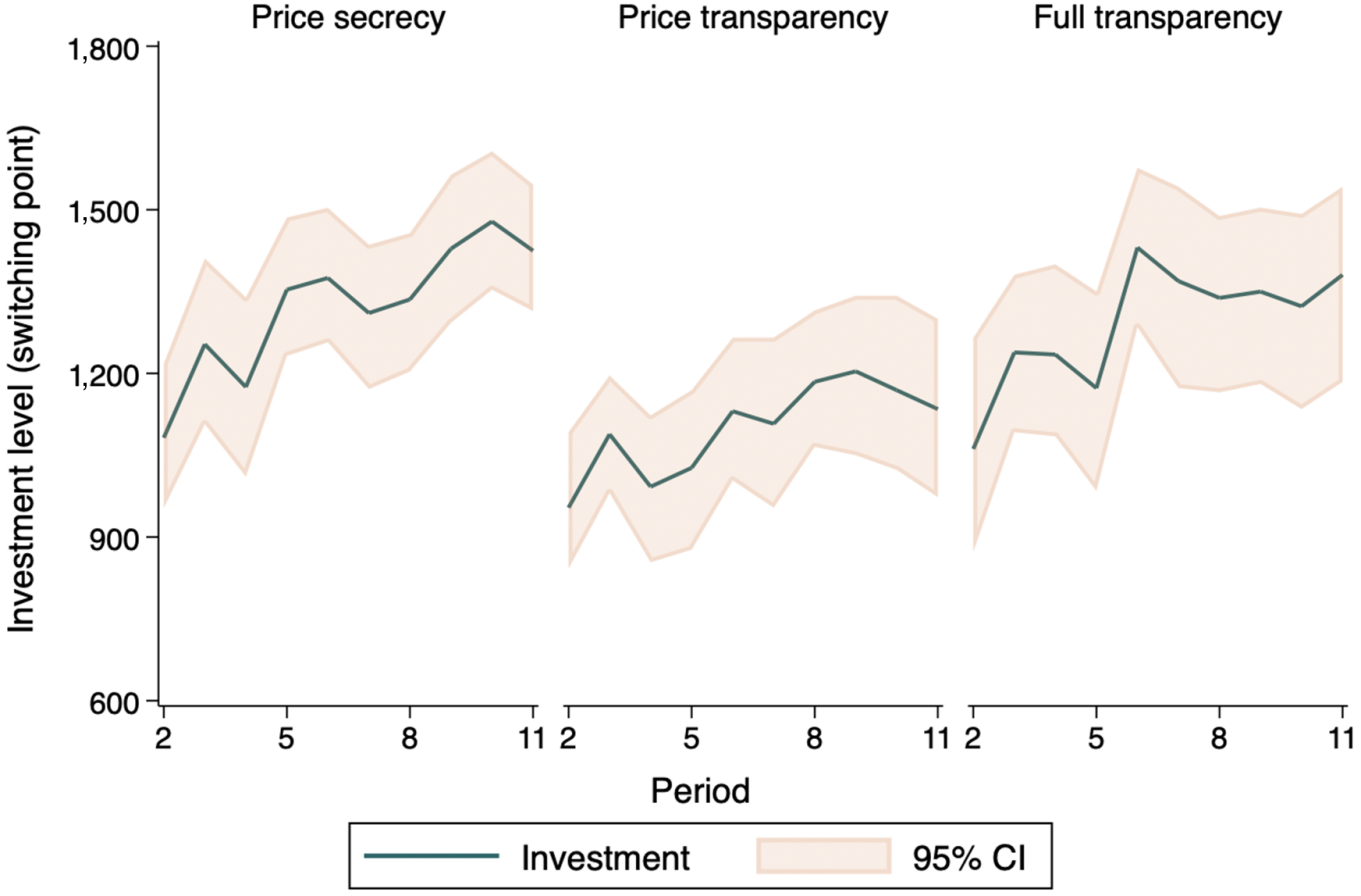
Average investment level in R&D, points per period. Notes: The lines represent the average maximum willingness to invest in R&D for the pharmaceutical company, by arm. Shaded areas are 95% bootstrapped confidence intervals.

Willingness to invest in R&D decreases significantly in *Price Transparency:* (A2: −16.86%; *P*: 0.002). Instead, we found no statistical difference in willingness to invest in R&D between *Price Secrecy* with *Full Transparency* (A3: −2.4%; *P*: 0.377). In Fig. 2, we show the evolution of average investment levels per arm over the 10 periods.

**FIGURE 3:**
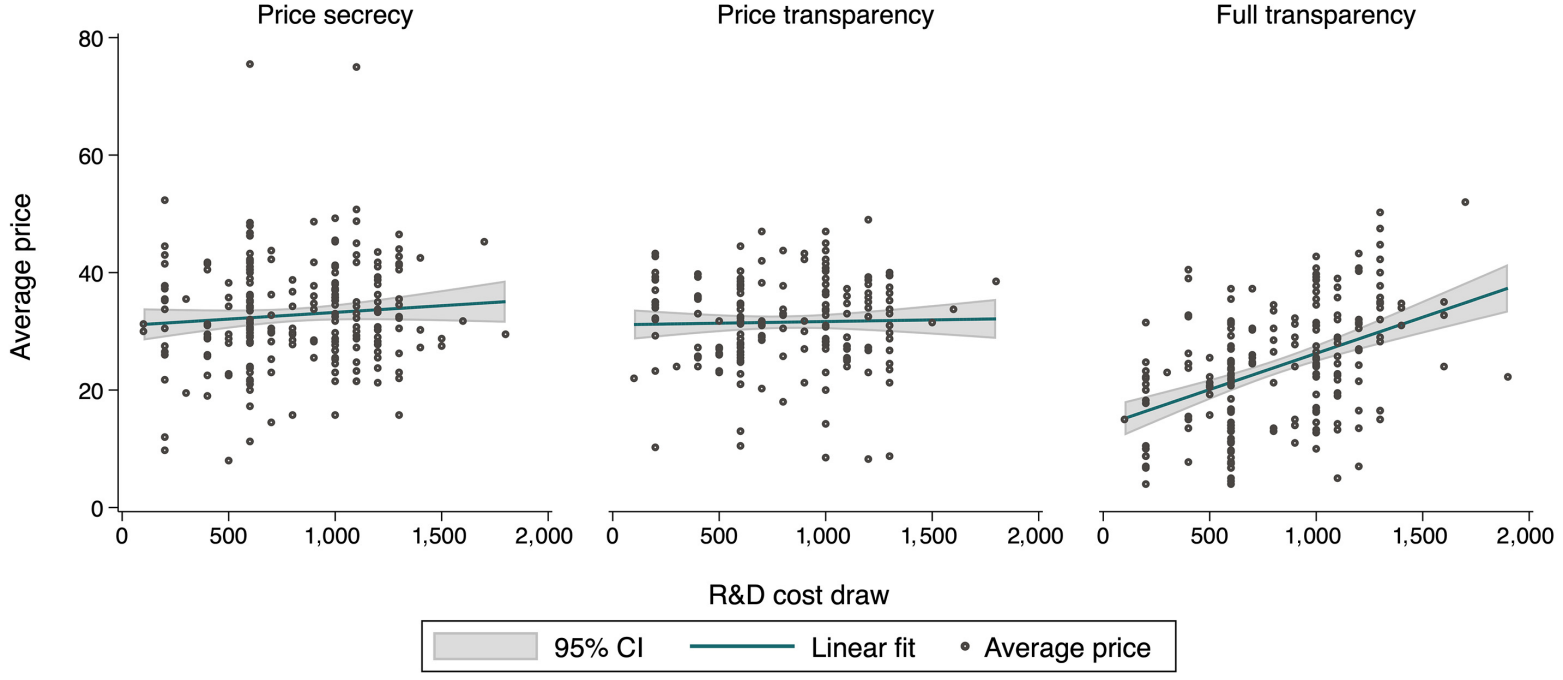
R&D cost factoring, in points. Notes: Average prices compared with R&D cost draws in a period, conditional on reaching agreement. Dots represent one cost draw—average price pair, lines are linear fits with 95% confidence intervals as the shaded area.

When comparing average prices with R&D cost draws over time, we see a weak positive correlation between average prices and R&D costs in *Price Secrecy* and no correlation in *Price Transparency*, when cost draws are only known to the pharmaceutical companies. Instead, making R&D costs transparent in *Full transparency* leads to a strong correlation between the two variables (Fig. 3).

### Individual and Aggregate Welfare

No statistical difference was found in aggregate welfare between *Price Secrecy* and *Price Transparency* (A2: −13.4%; *P*: 0.123) and between *Price Secrecy* and *Full Transparency* (A3: 6.9%; *P*: 0.511). Most of the difference in welfare between *Price Secrecy* and *Price Transparency* can be explained by the fact that the pharmaceutical company invests less, and countries lose the opportunity to treat patients in periods where there is no investment. In fact, conditioning on periods where the pharmaceutical company has invested, aggregate welfare is comparable between *Price Secrecy* and *Price transparency*.

**FIGURE 4:**
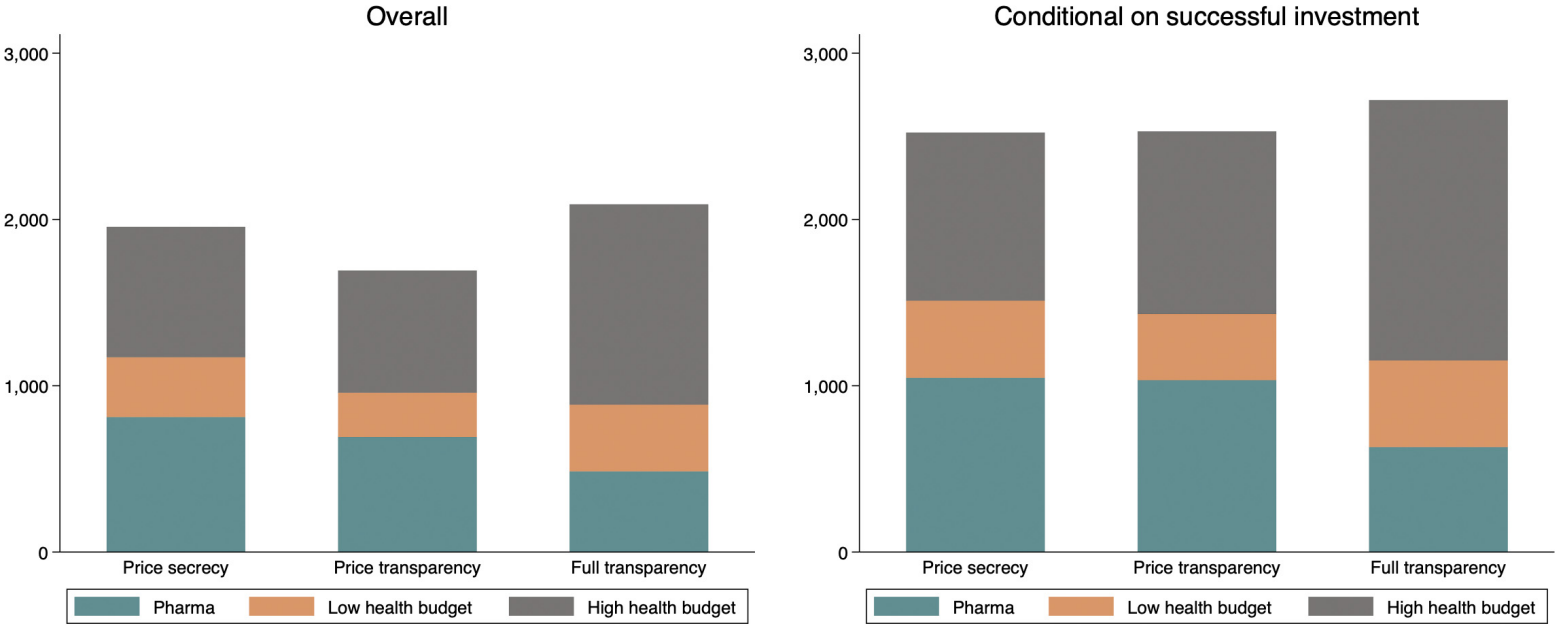
Individual welfare overall (left) and conditioned on successful investment (right), earnings per player per arm in points.

Figure 4 displays the company's welfare (i.e., profits) and the welfare of low- and higher-health-budget countries separately. We see that conditioning on successful investment (Fig. 4, right), welfare is very similar in *Price Secrecy* and *Price transparency*, while countries receive a larger share of the aggregate welfare in *Full Transparency*. In particular, the pharmaceutical company captures 45.2% of overall welfare in *Price secrecy*. This share remains similar at 44.6% in *Price transparency* (*P*: 0.917), but decreases strongly in *Full transparency* to 24.2% (*P* < 0.001).

In both experimental arms, the number of treated patients was statistically similar to *Price Secrecy* (*Price transparency* A2: −12.70%; *P*: 0.107; *Full Transparency* A3: 4.20%; *P*: 0.580).

## Discussion

Through a cross-country economic experiment, this study provides first evidence that combining medicine price and R&D cost transparency (*Full Transparency)* could be an effective policy to contribute to sustainable medical prices that balance affordability and investments in R&D. In the experiment, *Full Transparency* has performed surprisingly well from the perspective of countries: Aggregate welfare remained unchanged but was redistributed to their benefit. This originated from a 26% decrease in average prices. In spite of reduced industry profits, investment levels remained stable. In contrast, *Price Transparency* alone did not prove a promising policy to improve access to affordable treatments. It had no effect on overall average prices but led to price increases for lower-health-budget countries and reduced investments in R&D.

The pharmaceutical industry has raised concerns that price transparency would lead to reduced access, especially for lower-health-budget countries, who would no longer receive targeted discounts (16). We did not find evidence supporting concerns that countries are cutoff from negotiations. Countries reached agreements in virtually all negotiations (98.4%), with the lower-health-budget group not being excluded because of transparency. Arguably, this is because countries continue to accept that prices may differ based on purchasing power when these differences are made transparent. Prices converged only slightly as more transparency was introduced and seldom, if at all, at the expenses of access of the countries in the lower-health-budget group. In fact, access, as measured by the overall number of treated patients, was maximal under *Full transparency,* thanks to lower overall prices. However, when transparency concerned only prices, we could see some indication of price increases for lower-health-budget countries (+15% price increase; *P*: 0.051). This supports concerns of lower-health-budget countries and should be accounted for when evaluating policies that consider transparency of prices alone.

A second main finding is that price levels were unchanged in *Price Transparency*, suggesting that disclosure of net prices alone may not be sufficient to reduce prices effectively. This is in line with first evidence from transparency laws in Medicaid prescription spending, which also finds a limited effect (35). *Full Transparency,* on the other hand, was effective in lowering prices. This could stem from a change in what countries, and potentially also the company, consider to be an adequate price when R&D costs and the associated split of the surplus are made transparent. Presumably, countries realized the extent of industry profits and were able to negotiate for discounts especially in the case of low R&D costs. Besides fairness considerations, earlier economic experiments in other market settings, such as the Californian electricity markets, have found that removing a negotiator's informational advantage lower their bargaining power (36).

Another observation is concerned with the effect of transparency on R&D investments. The industry warns that transparency may create a downward pressure on prices. This in turn would reduce industry earnings and make R&D investments less attractive. Comparing empirical data on the R&D research intensity between Europe, subject to more price regulations, and the United States, subject to less price regulations, studies have been reporting a negative relationship between price regulation and R&D investments (37, 38). Concluded reasons are lagged cash flows and profit expectations (38). However, more recent evidence shows that this relationship does not persist when including company specific characteristics in statistical models, suggesting that investment choices are to a large extent explained by companies’ internal investment strategies and capabilities (39). In our experiment, we find a surprising non-monotonic effect of transparency on R&D investments. Investment decreased significantly in *Price Transparency*. However, original investment levels could be restored in *Full Transparency*. This pattern is not supported by the price dynamics because prices were actually lowest in *Full Transparency*.

It is a surprising finding, challenging economic intuition. One explanation could lie in the stark difference in the extent to which prices correlate with R&D costs across experimental arms. As shown in Fig. 3, *Full Transparency* has led to prices that closely track R&D costs. Thus, pharmaceutical companies could be reassured to recover high R&D costs through high prices—even in the absence of contracts binding countries to offer such coverage ex-ante. This result is in line with findings from prior economic experiments (40). Shared risk between countries and the company could have helped to sustain R&D investments despite lower overall prices. Thus, under *Full transparency*, prices seem to track both R&D costs (with higher prices associated to higher R&D costs) and valuations (with larger prices paid by countries with higher willingness-to-pay). From an economic viewpoint, and to the extent that costs are not strategically misreported or manipulated, such a price determination system is efficient in that it gives the right incentives to develop medicines with the highest return to investment. It also offers pharmaceutical companies the opportunity for risk sharing, which is necessary especially if they face reduced margins brought about by lower prices. This might give justification to reimbursement models that go beyond pure added therapeutic value but also consider R&D and/or production costs to determine a fair price of a medicine (41–44). Our evidence further reveals a rather weak positive correlation between prices and R&D costs in *Price Secrecy* (see Supplementary Data S3, column 1). There, even though R&D costs are not public, the pharmaceutical company manages to a limited extent to recover higher R&D costs with higher prices. This correlation disappears entirely in *Price Transparency*, possibly because higher-health-budget countries observe that lower-health-budget countries get access to the medicine at a discount, which may make it harder to convince countries to reimburse high R&D costs. The absence of risk sharing may then have diminished pharmaceutical companies’ willingness to invest in *Price Transparency*.

Notice that high prices in support of high R&D costs may be considered fair by participants in all experimental arms. Pharmaceutical companies was not able to credibly communicate them when these costs were not made public by design, and in particular so in *Price Transparency*. Interestingly, the decision to invest less in *Price Transparency* was not in the best interest of the company, which at the observed prices would have made larger profits with more investment. In fact, prices were still significantly higher than in *Full Transparency* and, although rigid, large enough to recover high R&D costs. Besides aversion to bearing the full risk of high costs, a complementary explanation is that the pharmaceutical company may hold incorrect beliefs regarding the chances to recover high costs. Such beliefs can be self-reinforcing: As companies never develop medicines when R&D costs are high, they never learn that these costs can be recovered. Our results suggest that the link between price regulation and R&D investments is more complex than a simple monotonic relationship between the two, calling for more research in this area.

A key point of discussion is how generalizable and transferable the results of our laboratory experiments are to the real world. Naturally, such an experiment has to build upon a simplified model of reality, only including variables that are most important to the research question at hand. For example, we did not include variations in clinical effectiveness in the experiment, as we did not expect them to have an impact on the effect of transparency. Furthermore, we limited the experiment to four buying countries, a choice that could have limited potential effects of strategic launch sequences. It however allowed us to collaborate with laboratories in the respective countries, capturing realistic cultural dynamics that might influence the acceptance of price differences. It is important to emphasize that an advantage of our study, and of controlled experiments in general, is standardizing external factors, minimizing their effect on the results. The experiment was conducted using state-of-the-art protocols for economic experiments and a standard sample of student populations. There is robust evidence that intervention effects found in these samples generalize to other samples and the field (25, 26). Laboratory experiments have proven to be a major source of knowledge in the social sciences, and human behavior in the laboratory usually correlates with real-world behavior (45). As a result, laboratory experiments are often used to inform policy making, for example in the domain of spectrum auctions (46). We regard our empirical evidence as proof of principle. We see policy pilots as the natural next step in the exploration of the effect of transparency, and, based on our findings, recommend that R&D cost transparency be considered jointly with price transparency.

However, technical difficulties in ensuring truthful reporting of R&D costs must not be underestimated. R&D costs are difficult to isolate for a single project, must account for the cost of failed candidates, and a policy based on R&D cost transparency bears the risk of companies inflating R&D costs. As a study on creative compliance in the presence of profit controls shows, profit controls may lead to inefficiencies or an inflation of reported costs (47). While our experiment prevented the pharmaceutical company from strategically misreporting R&D costs in *Full transparency*, crucially it also showed that the company failed to credibly communicate their costs in the other two experimental arms, as suggested by the fact that prices remained largely unresponsive to them. Credible and verifiable communication of these costs could benefit both the countries, who would be protected from strategic misreporting, and the pharmaceutical companies, who could be able to effectively share the risk of high R&D costs. After all, transparency could be seen as a more favorable option for pharmaceutical companies than rigid profit and price controls because it offers more flexibility to respond to product-specific factors in negotiations. A voluntary reporting system similar to the one some companies follow for direct-to-consumer prices (48) is therefore at least possible.

Our results highlight that it is imperative to extensively explore technical options that lead to a truthful and accessible reporting of R&D cost. For example, public reporting systems similar to those in place in the financial sector, and opportunities of blockchain technology to track costs, as it is suggested to improve transparency in the pharmaceutical supply chain (49), could be considered. A first step can lie in the rigorous disclosure of public R&D investments. Better access to the evaluations that accompany the frequent mergers and acquisitions in the biotechnology sector could further help to assess financial streams. Engaging with stakeholders such as regulators, investors, and the industry is the important next step to contextualize these empirical findings and to explore how R&D cost and price transparency could be technically implemented.

To conclude, we conducted a first in the field economic experiment to generate evidence on policy proposals to reduce drug costs at market launch. Our findings suggest that *Price transparency* in itself may not add to affordability. However, combining drug price and R&D cost transparency could be an effective policy to contribute to sustainable medical treatment prices that balance affordability and sustained investments in R&D. The relationship between drug pricing policies and R&D investments might be more complex than a simple linear model in which lower average prices lead to lower investments. In particular, transparency and verifiability of R&D costs lead to prices that, albeit lower in general, more closely track the R&D costs—thus offering the pharmaceutical companies reassurance that their costs will be recovered. More research is needed to explore options to effectively implement R&D cost transparency and to understand factors influencing the investment decisions of companies.

## Supplementary Material

Supplementary Data File 3 Detailed results.

Supplementary Data File 1 Detailed methods.

Supplementary Data File 2 Experimental instructions.
