# Supplementary material for: Affordable Prices Without Threatening the Oncological R&D Pipeline—An Economic Experiment on Transparency in Price Negotiations
Source: Cancer Res Commun. 2022 Jan 27;2(1):49–57. doi: 10.1158/2767-9764.CRC-21-0031 (PMC9973423; doi:10.1158/2767-9764.CRC-21-0031)
Supplement: Supplementary Data File 3 — Detailed results. [file crc-21-0031-s01.docx]

**Supplementary information S 3 - Extended results**

Corresponding to Figure 4, Table 1 presents how average prices respond R&D cost draws. We regress the average price in each market on R&D cost draws as well as some controls based on our questionnaire preference measures. (1) to (3) present results for each treatment separately, (4) and (5) present results for the pooled data. In (4) and (5), the baseline is *Price secrecy*, and experimental arm dummies interacted with R&D costs indicate differential effects across treatments. We observe that prices most closely track R&D costs in *Full transparency*.

Table S3: R&D cost factoring

|  | (1) | (2) | (3) | (4) | (5) |
| --- | --- | --- | --- | --- | --- |
|  | Price secrecy | Price transparency | Full transparency | Pooled | Pooled |
| R&D costs | 0.00231+ | 0.000603 | 0.0137* | 0.00314+ | 0.00323* |
|  | (0.00125) | (0.00126) | (0.00169) | (0.00166) | (0.00156) |
|  |  |  |  |  |  |
|  |  |  |  |  |  |
| Price transparency |  |  |  | 0.236 | 2.159 |
|  |  |  |  | (2.814) | (2.679) |
|  |  |  |  |  |  |
| Full transparency |  |  |  | -17.02* | -14.47* |
|  |  |  |  | (3.090) | (2.949) |
|  |  |  |  |  |  |
|  |  |  |  |  |  |
| Price transparency # R&D costs |  |  |  | -0.00195 | -0.00223 |
|  |  |  |  | (0.00213) | (0.00197) |
|  |  |  |  |  |  |
| Full transparency # R&D costs |  |  |  | 0.00999* | 0.00958* |
|  |  |  |  | (0.00256) | (0.00225) |
|  |  |  |  |  |  |
|  |  |  |  |  |  |
| Period | -0.288 | -0.334* | -0.981* | -0.512* | -0.513* |
|  | (0.184) | (0.159) | (0.168) | (0.103) | (0.103) |
|  |  |  |  |  |  |
| Countries' average altruism | -0.782 | 1.902 | -1.371 |  | 0.235 |
|  | (2.268) | (1.387) | (1.539) |  | (1.026) |
|  |  |  |  |  |  |
| Countries' average risk aversion | -0.833 | 0.863 | 1.574 |  | 0.382 |
|  | (1.275) | (0.981) | (1.716) |  | (0.912) |
|  |  |  |  |  |  |
| Countries' average competitiveness | -0.885 | 0.155 | -2.439+ |  | -0.729 |
|  | (1.621) | (0.986) | (1.278) |  | (0.921) |
|  |  |  |  |  |  |
| Company's risk aversion | 0.972 | -0.414 | 1.635 |  | 0.726 |
|  | (0.921) | (0.724) | (1.160) |  | (0.487) |
|  |  |  |  |  |  |
| Company's altruism | -1.504* | 0.560 | -2.177* |  | -1.133* |
|  | (0.649) | (0.617) | (0.694) |  | (0.372) |
|  |  |  |  |  |  |
| Company’s competitiveness | -1.450* | 1.110 | -0.513 |  | 0.0223 |
|  | (0.686) | (0.698) | (0.946) |  | (0.497) |
|  |  |  |  |  |  |
|  |  |  |  |  |  |
|  |  |  |  |  |  |
| Constant | 61.28* | 5.318 | 47.40* | 33.70* | 36.77* |
|  | (19.49) | (11.77) | (17.95) | (2.295) | (11.62) |
| Observations | 217 | 173 | 199 | 589 | 589 |

*Notes:* The dependent variable across all specifications is the average price paid in the market by the countries. (1) to (3) regress prices on R&D costs as well as questionnaire measures of preferences for the average across all countries, and the company separately, for each experimental arm. (4) and (5) pool all data and use experimental arm dummies as well as experimental arm dummies interacted with R&D costs to test for differences between arms. Standard errors clustered at the group level in parentheses. ^+^ *p* < 0.10, ^*^ *p* < 0.05.
