# Supplementary material for: Affordable Prices Without Threatening the Oncological R&D Pipeline—An Economic Experiment on Transparency in Price Negotiations
Source: Cancer Res Commun. 2022 Jan 27;2(1):49–57. doi: 10.1158/2767-9764.CRC-21-0031 (PMC9973423; doi:10.1158/2767-9764.CRC-21-0031)
Supplement: Supplementary Data File 1 — Detailed methods. [file crc-21-0031-s02.docx]

**Supplementary information - S1**

**Market game and parameters**

In the following, we describe the set-up of the market game and the main parameter choices. Figure 1 gives an overview of the phases of the experiment.

**Figure S1 - Experimental timeline**


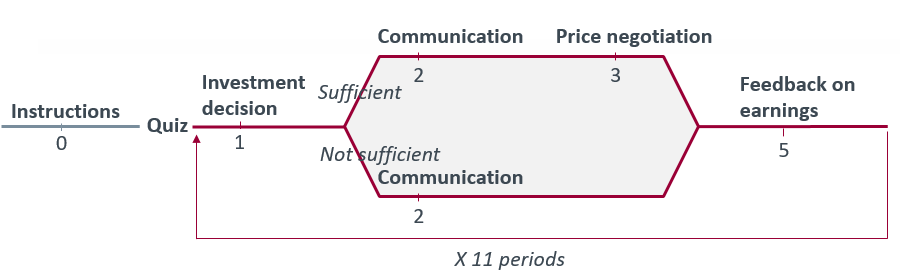


**0) Instructions and Quiz**

At the beginning of the experiment, participants were presented with the experiment instructions (supplementary information 2) and a quiz to test their understanding. Only after passing the quiz, they were allowed to move to the experimental phase. The experimental phase started with one learning period that was not included in the main analysis.

**1) Investment decision:** The pharmaceutical company first decided its maximal willingness to invest in R&D. For this purpose, the company faced a multi-price list: For each potential R&D cost between 100 and 2000, it decided whether it would be willing to invest for this cost. After this choice, the pharmaceutical company learned the randomly determined R&D costs, drawn from a discretized truncated normal distribution with mean 850 and standard deviation 550. This distribution was unknown to participants, and the draws were *iid* per period and across groups. ^[[1]](#footnote-1)^ If the company had indicated that it was willing to invest for the randomly drawn R&D-costs in that period, it paid the drawn cost and a medicine was developed. The company and the countries then entered a market and negotiated about the price of the developed medicine. If for the randomly drawn costs, the pharmaceutical company decided not to invest, no medicine was developed and negotiations would not occur with each participants keeping their initial budget.

**2) Communication:** While part of our markets rely on structured bargaining within the double auction, we also allowed participants to communicate using text messages within the experimental software. Again, this likely matches the real-world interaction, but may also facilitate a better understanding of the problem at hand by letting participants discuss while the experiment proceeds.(1) Following the investment choice, participants had the opportunity to communicate among each other in three free-form communication channels using text messages for 3.5 minutes. These communication channels included: one with all participants, one all countries (excluding the pharmaceutical company), and one private bi-lateral channel between each country and the company. The communication phase was available also when no investment took place. In this latter case, following this chat, all players moved to the next period, following an identical structure.

**3) Price negotiation:** Following the drug development, a bargaining stage (double auction market) opened in which countries and the company could exchange per-unit price offers.

**4) Feedback on earnings:** In a last step, the participants received feedback on their earnings. The structure of this earnings was as follows: In each period, each country has an initial budget (total amount of money it can spend), a total number of eligible patients (each needing one unit of the medicine), and a maximal willingness-to-pay for each patient representing the value the country earns by treating each patient who receives a medicine. Thus, countries’ earnings if there was a successful investment in R&D and successful price negotiations are determined as follows:

($ Country budget)

+

[(# patients served with medicines) x ($ willingness-to-pay per patient)]

–

[(# patients served with medicines) x ($ agreed price)]

In each period, the pharmaceutical company has an initial budget of 1000 points. If investment is successful, the pharmaceutical company pays the R&D costs and can sell any number of units after paying per-unit production costs of one point.

Thus, payoffs for the pharmaceutical company are given by:

($ Pharma Budget) – ($ R&D costs)

+

∑^*^{[($ agreed price) – ($ production cost)] x (# patients served with medicines)}

^*^Sum across countries with which an agreement was reached.

To capture the importance of timely agreements, a time penalty of 10% of a country’s budget was imposed for each minute in which an agreement was not reached.

To increase the representativeness of the study, the outcomes of the negotiations were also tied to a small real-world impact on cancer patients. The number of fictitious medicines purchased determined the level of donations to cancer centers in each of the corresponding countries. Donations were 5 points for each medicine sold and thus proportional to the number of fictitious patients served.^[[2]](#footnote-2)^

**Experimental implementation**

In total, the market game consisted out of 11 periods. The total number of periods was not pre-announced to participants, to prevent endgame effects. To facilitate learning, the first period featured mandatory investment by the company. This period is dropped from the analysis, in accordance with the pre-registration plan.

All countries were represented by participants from experimental laboratories located in the countries they represented. For Poland, participants were registered at the Experimental Economics Lab at the University of Warsaw. For Spain, participants were registered at the LINEEX Lab at the Universidad de València. For the Netherlands, participants were registered at the CREED laboratory at the University of Amsterdam. For Germany and participants for the pharmaceutical company, participants were registered at the MELESSA lab at the LMU München. The standard participant pool from these laboratories were used, and standard protocols in experimental economics were employed, such as no deception, anonymity of participants and use of monetary incentives. Due to COVID restrictions, these participants joined the experiment in remote, from an environment of their own choice, often their home. At the moment of the study, all laboratories had developed protocols to run experiments in remote with their subject pool, and we followed the procedure each laboratory used.^[[3]](#footnote-3)^

Participants were on average 23 years old, and 240 out of 400 participants were female. Sessions lasted approximately two hours. In the experiment, participants could earn points. After concluding the experiment, each 3 points were exchanged for 2 Eurocents (or 3 Polish zloty in the case of Poland). Average and minimum earnings were dependent on the requirements of the laboratories. If necessary, we paid an unannounced bonus for completing the ending questionnaire for payments to match the laboratories’ targets (9 Euros in Spain, 5 Euros in the Netherlands, 18 PLN in Poland).

Each subject was paid the outcomes associated to two randomly selected periods and a laboratory specific participation fee (or minimum earnings) at the end of the experiment.

**References**

1. Brandts J, Cooper DJ, Rott C. Communication in laboratory experiments. Handbook of research methods and applications in experimental economics: Edward Elgar Publishing; 2019.

1. . Due to a coding error, the first 13 groups across all treatments all faced identical R&D cost draws in the same order. [↑](#footnote-ref-1)
2. For the Spanish donations, we transferred the donations to another center in Spain than originally communicated. This was announced together with the proof of the donation on our website. [↑](#footnote-ref-2)
3. In all locations, participants’ identity was verified based on laboratory policies, such as using a zoom meeting run by assistants not connected to the experiment itself. Payments were transferred using paypal or (anonymized) bank transfers. [↑](#footnote-ref-3)
