# Supplementary material for: Affordable Prices Without Threatening the Oncological R&D Pipeline—An Economic Experiment on Transparency in Price Negotiations
Source: Cancer Res Commun. 2022 Jan 27;2(1):49–57. doi: 10.1158/2767-9764.CRC-21-0031 (PMC9973423; doi:10.1158/2767-9764.CRC-21-0031)

**Supplementary information – S2**

**Page 1 – all experimental arms – all participants**


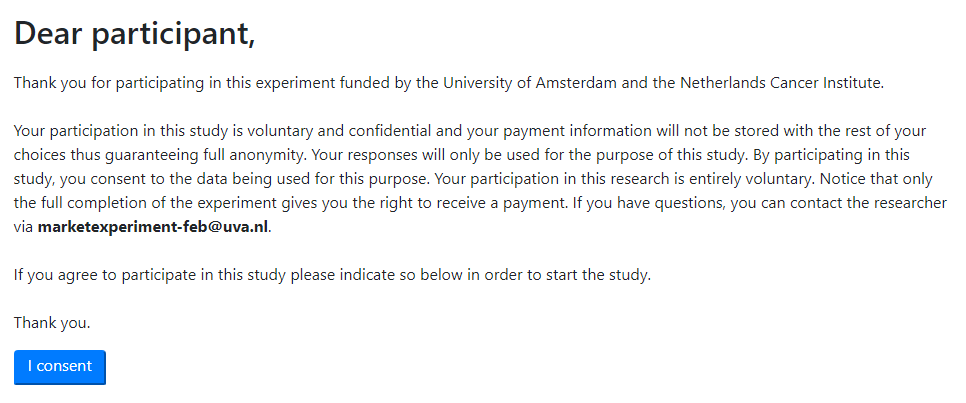


**Page 2 – all experimental arms – all participants***

*Note: exchange rates of points/local currencies differed between countries.


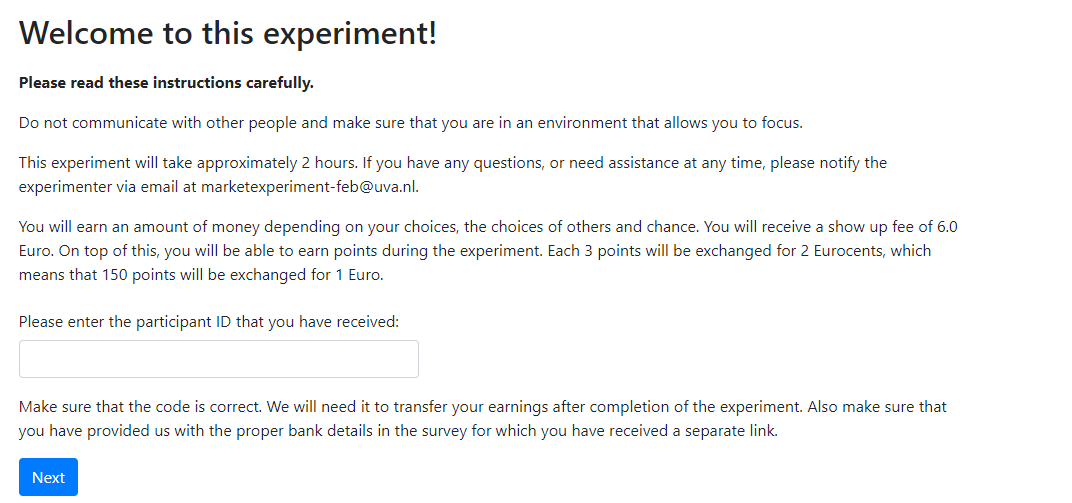


**Page 3 - all experimental arms – pharmaceutical company**


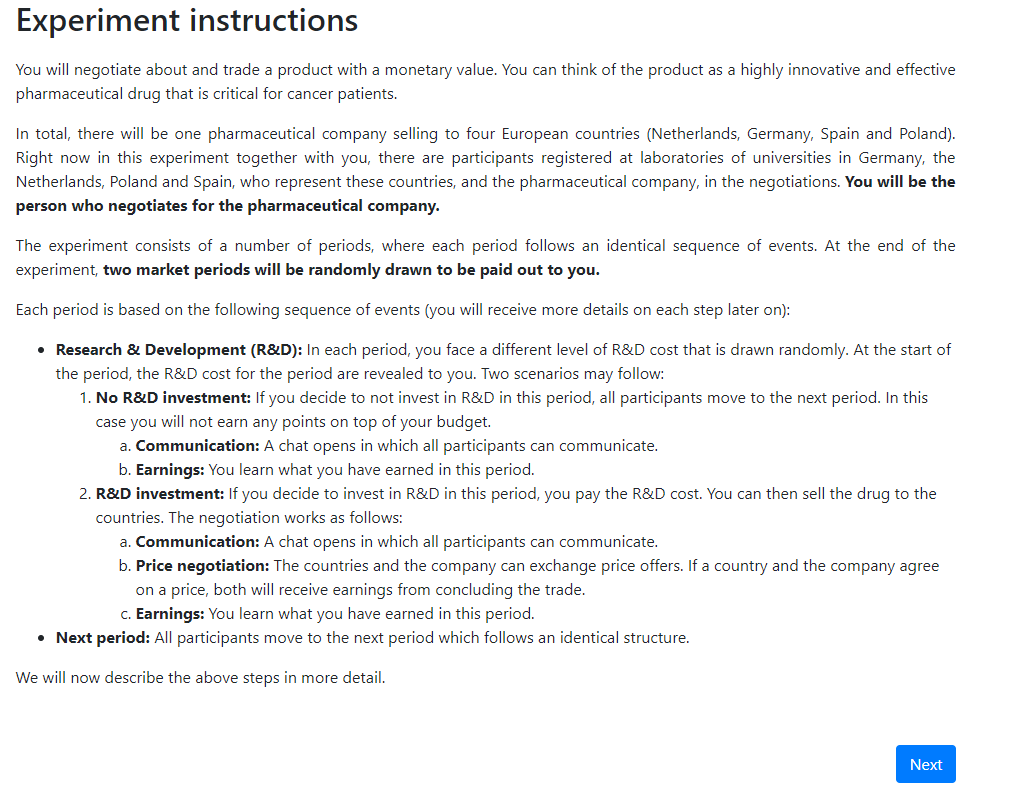


**Page 3 – all experimental arms - NL**


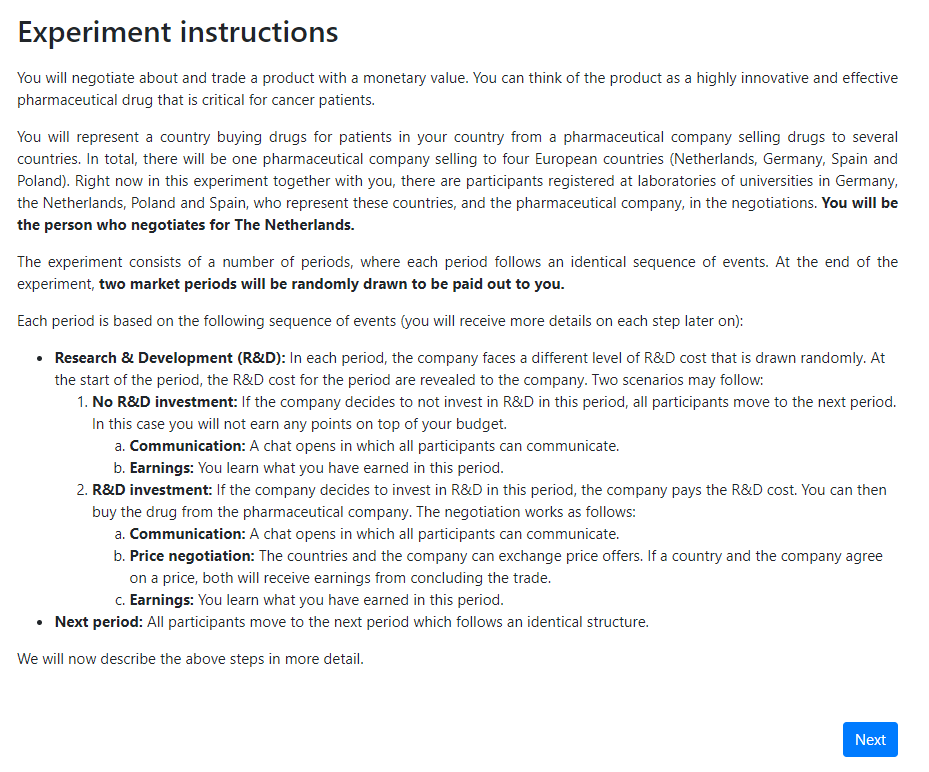


**Page 4 – experimental arm 1 “Price Secrecy” - Pharmaceutical company**


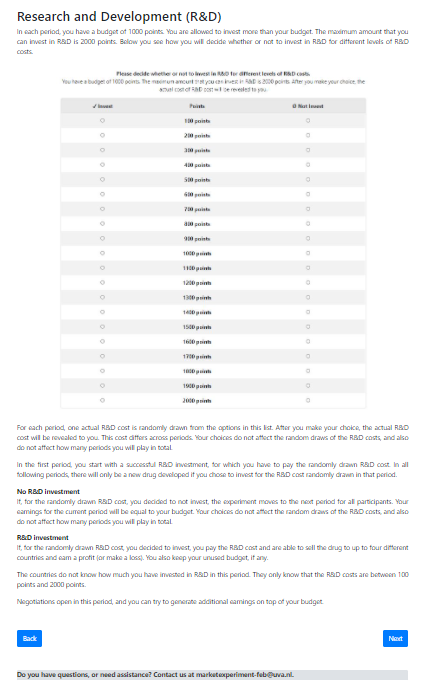


**Page 4 – experimental arm 2 “Price transparency” – Pharmaceutical company**


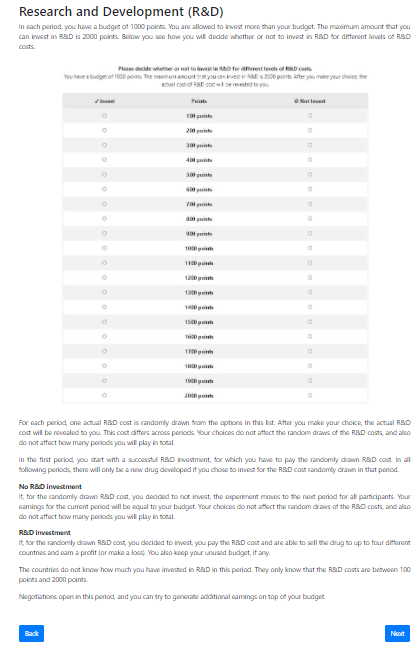


**Page 4 – experimental arm 3 “Full transparency” – Pharmaceutical company**


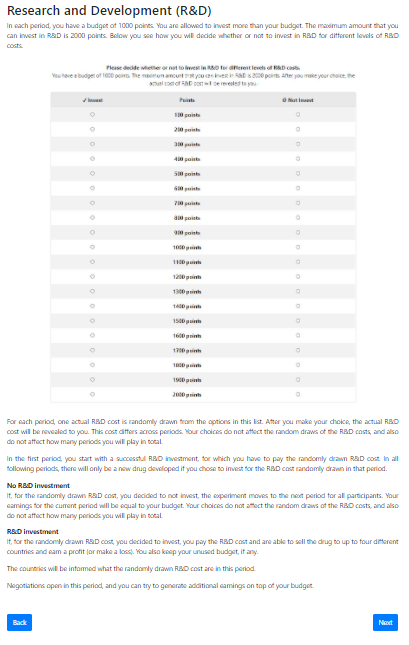


**Page 4 – experimental arm 1 “Price Secrecy” – NL**


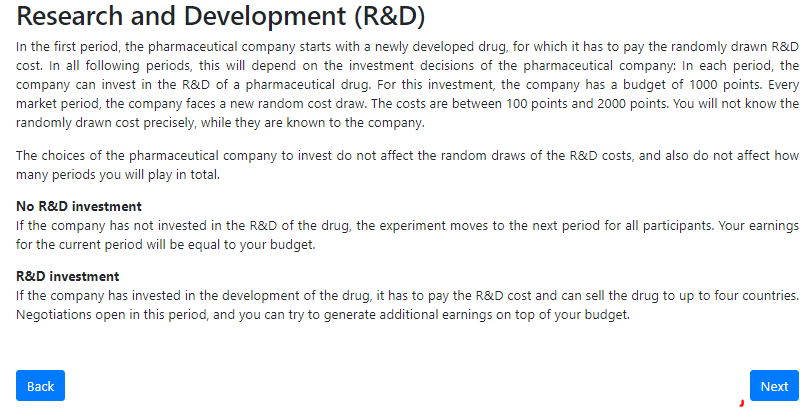


**Page 4 – experimental arm 2 “Price transparency” – NL**


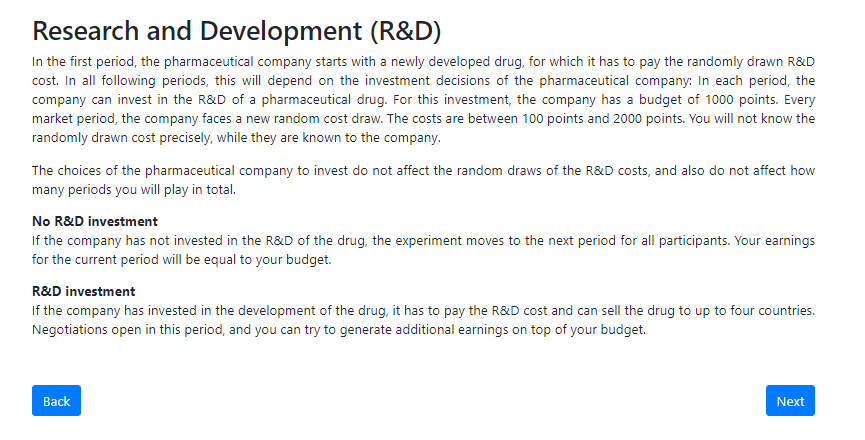


**Page 4 – experimental arm 3 “Full transparency” – NL**


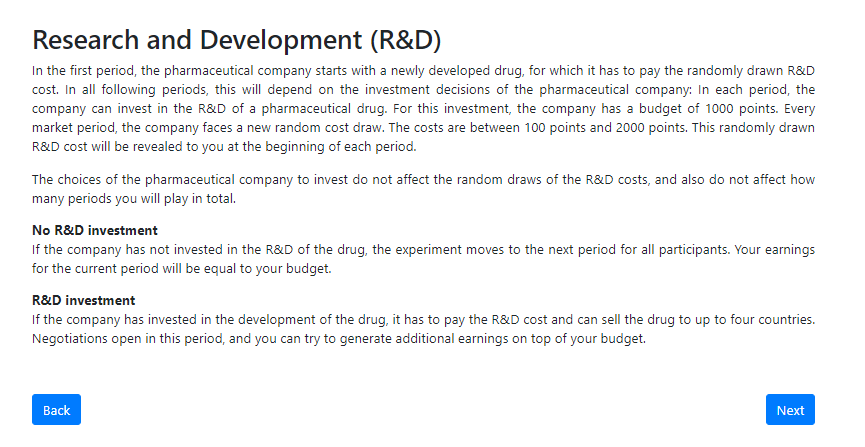


**Page 5 – experimental arm 1 “Price Secrecy” - Pharmaceutical company**


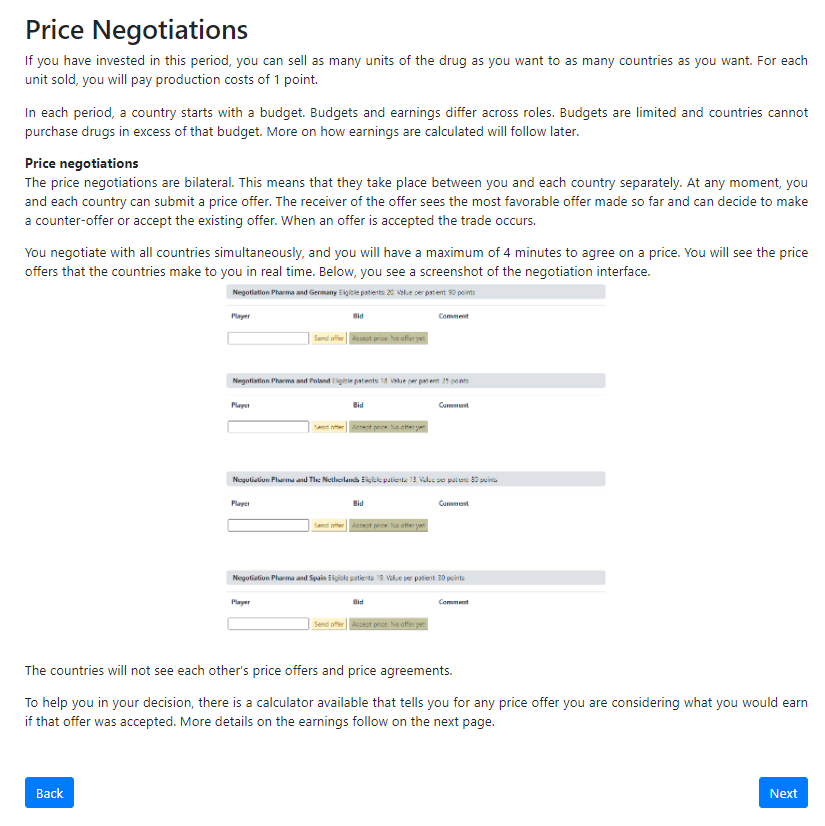


**Page 5 – experimental arm 2 “Price transparency” – Pharmaceutical company**


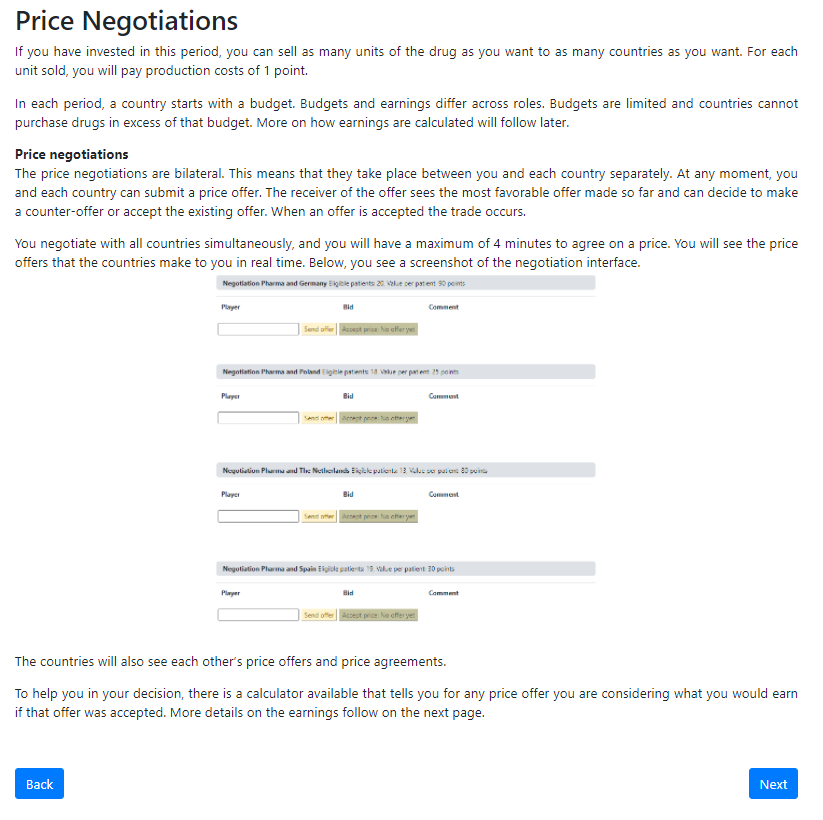


**Page 5 – experimental arm 3 “Full transparency” – Pharmaceutical company**


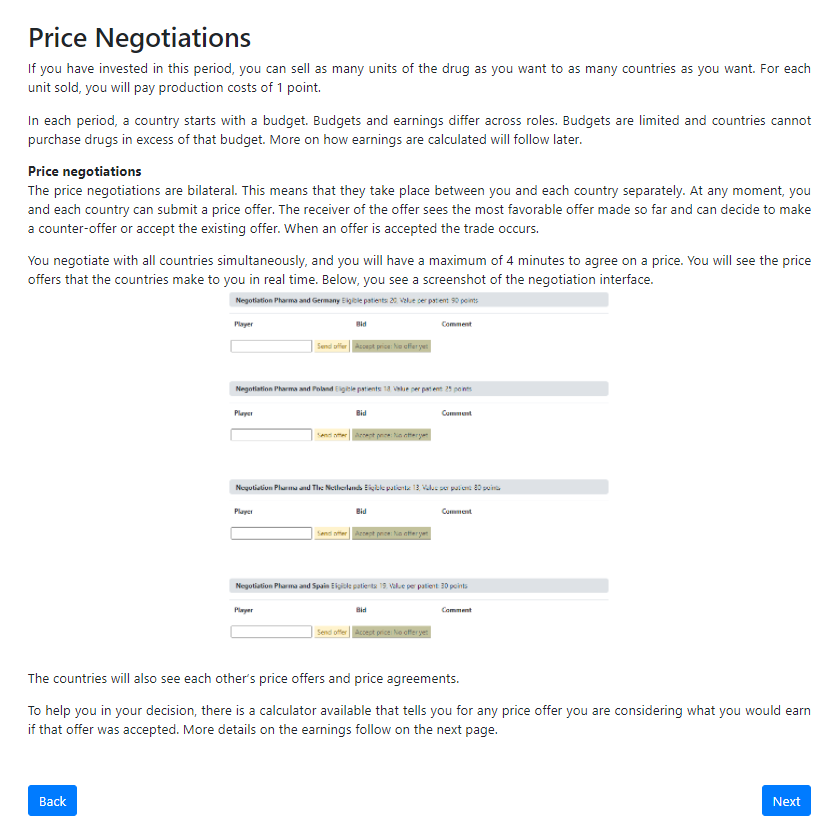


**Page 5 – experimental arm 1 “Price Secrecy” – NL**


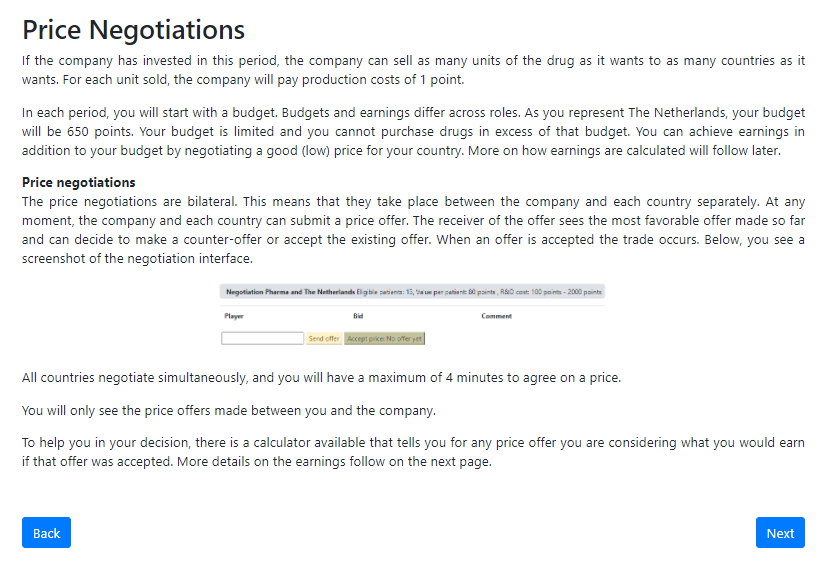


**Page 5 – experimental arm 2 “Price transparency” – NL**


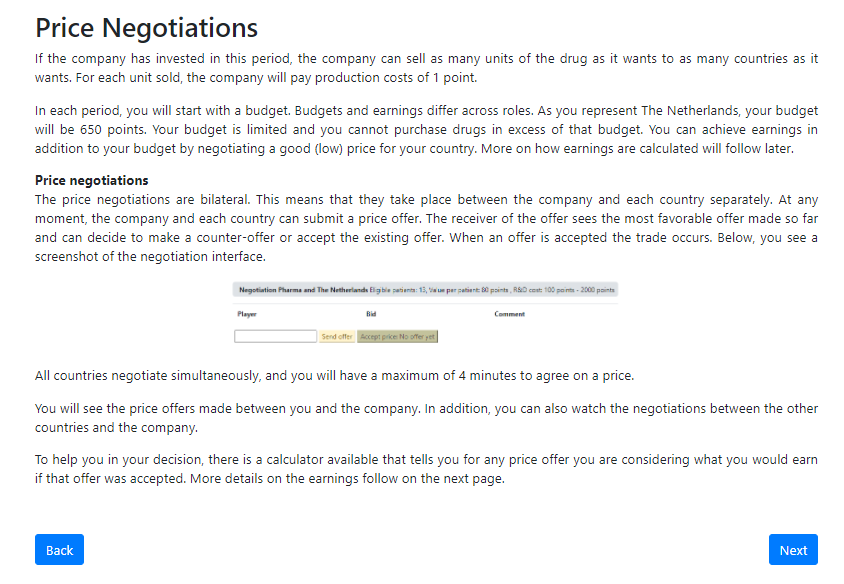


**Page 5 – experimental arm 3 “Full transparency” – NL**


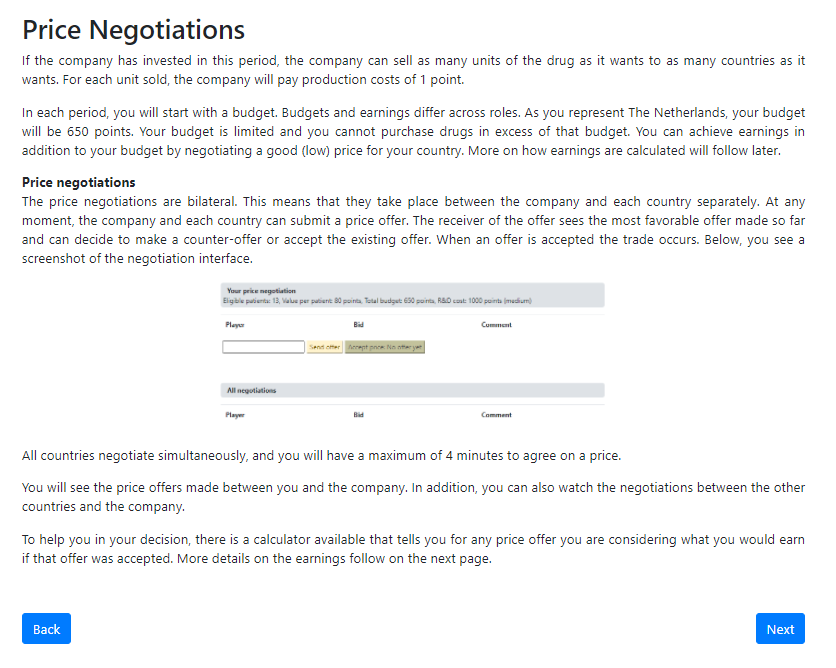


**Page 6 – All experimental arms - Pharmaceutical company**


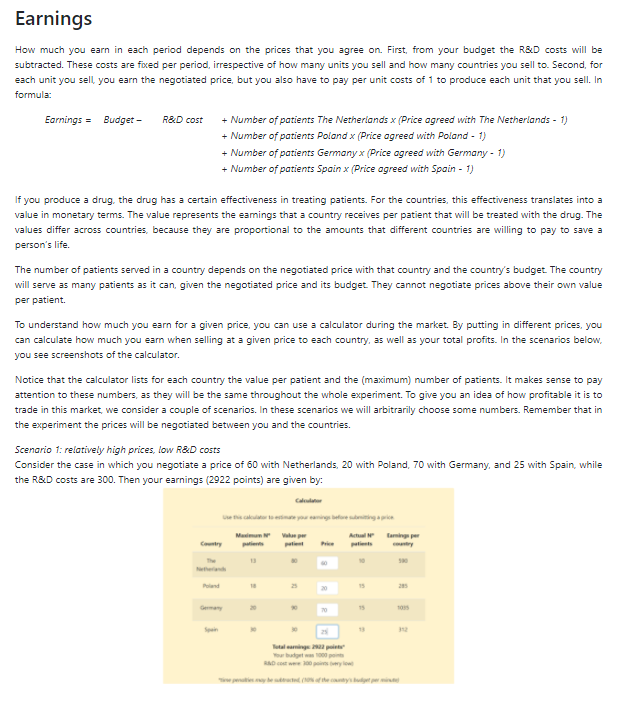


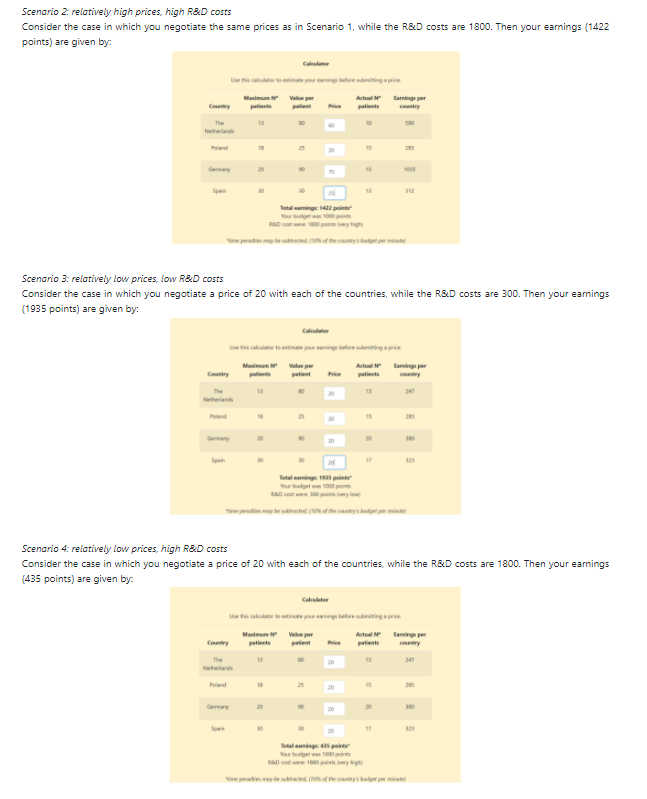

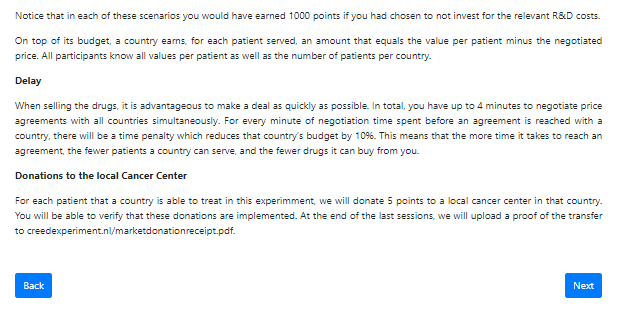


**Page 6 – All experimental arms – NL***

*Note: only the screenshots of the calculator differed between experimental arms with regards to displayed R&D costs.


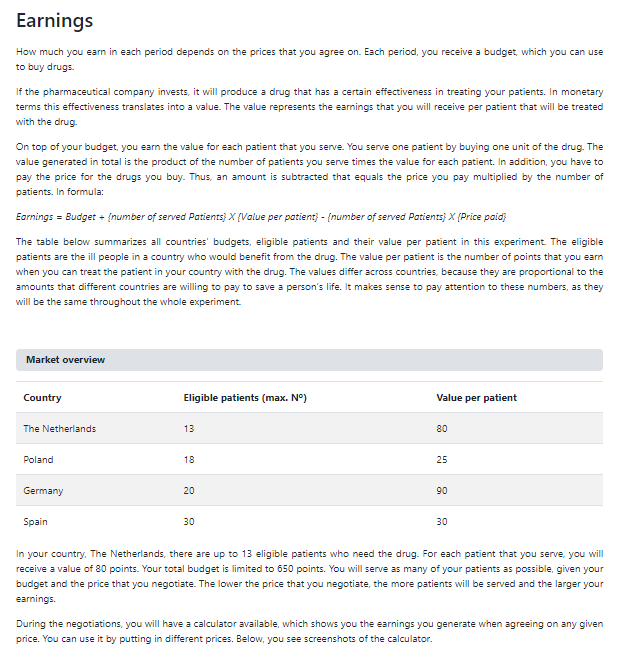


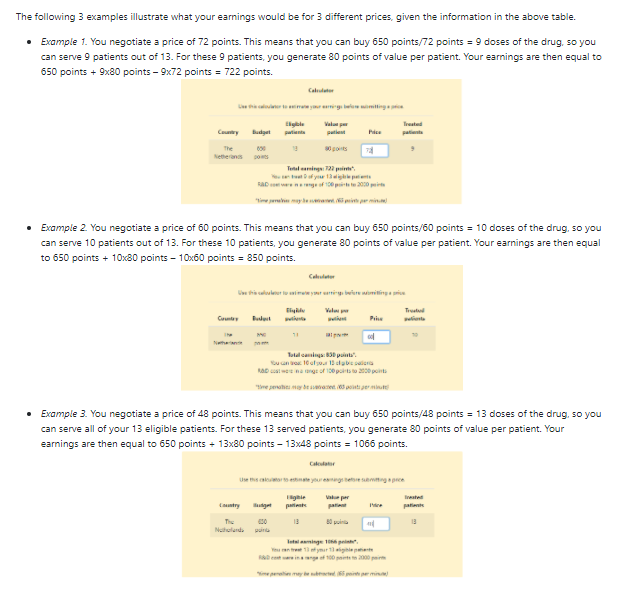

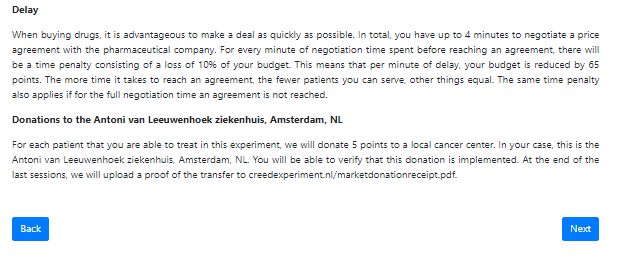


**Page 7 – experimental arm 1 “Price Secrecy” - Pharmaceutical company**


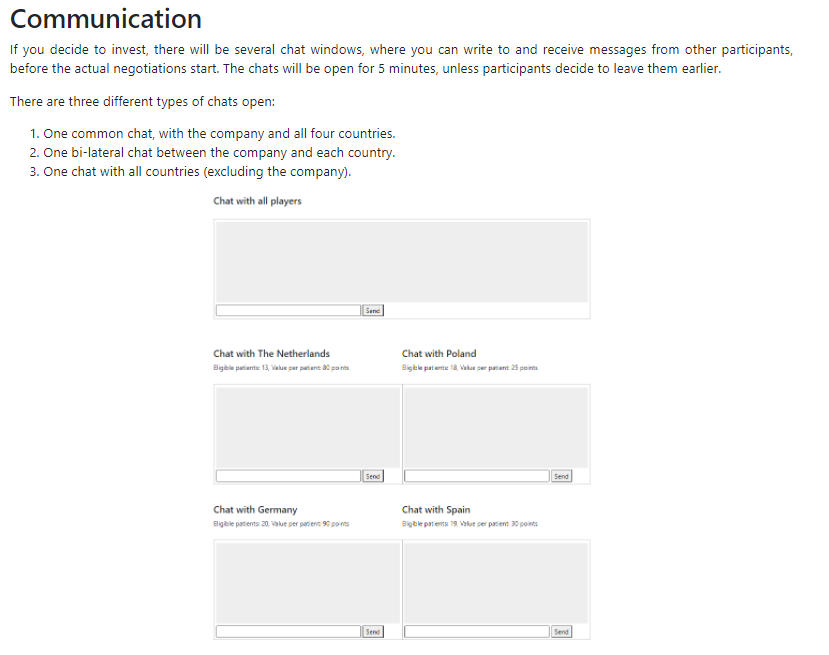


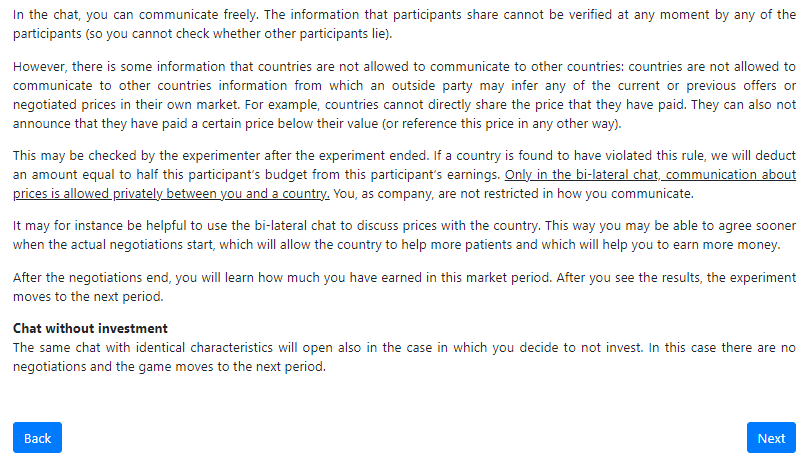


**Page 7 – experimental arm 2 “Price transparency” – Pharmaceutical company**


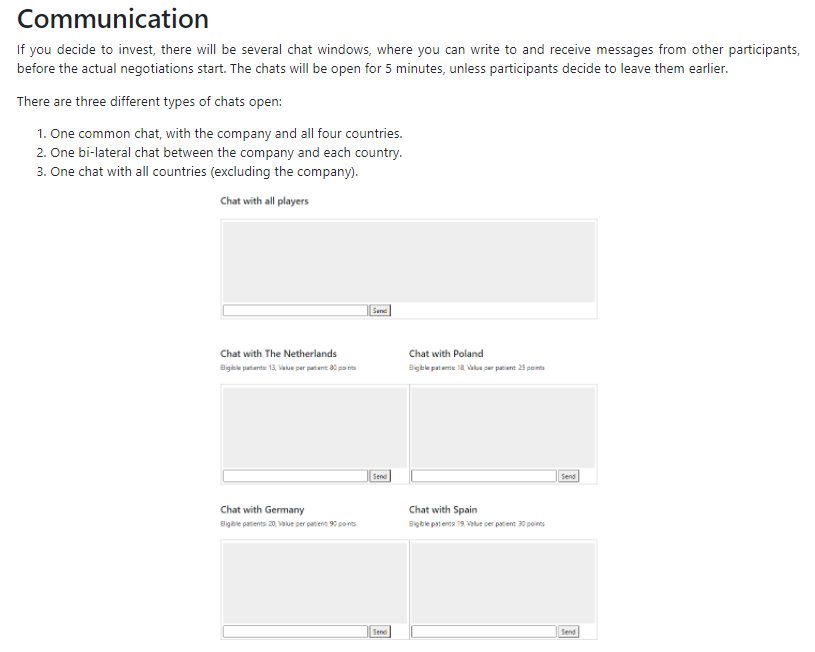


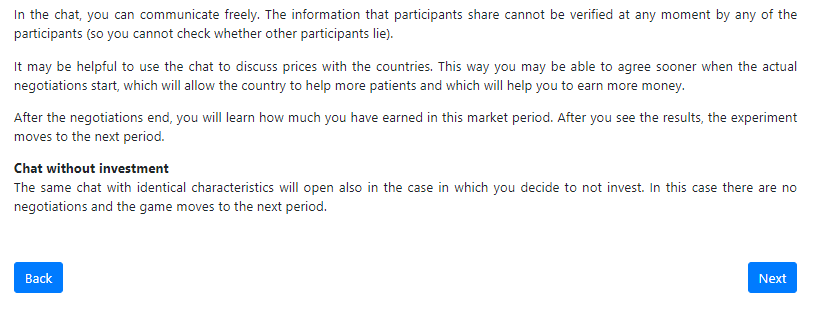


**Page 7 – experimental arm 3 “Full transparency” – Pharmaceutical company**


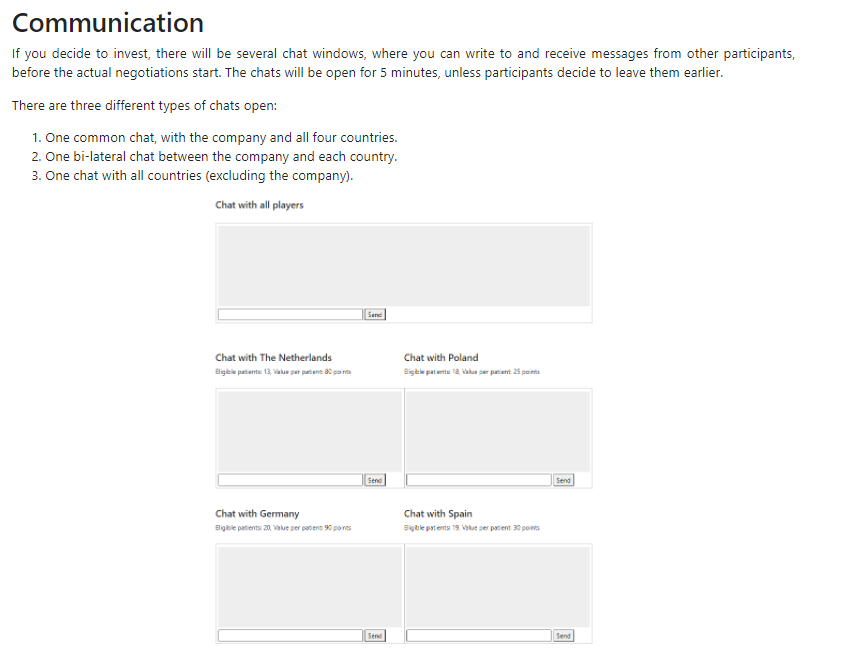


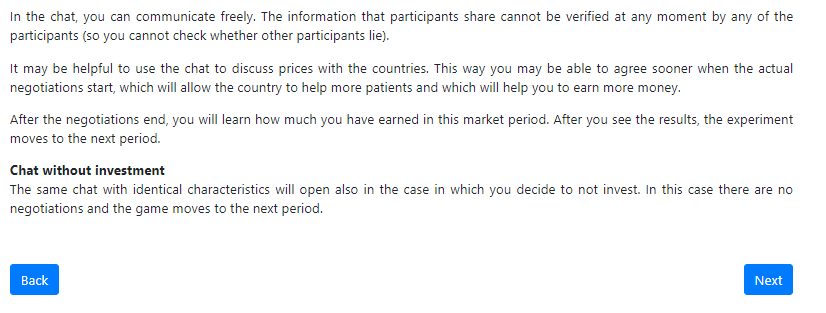


**Page 7 – experimental arm 1 “Price Secrecy” – NL**


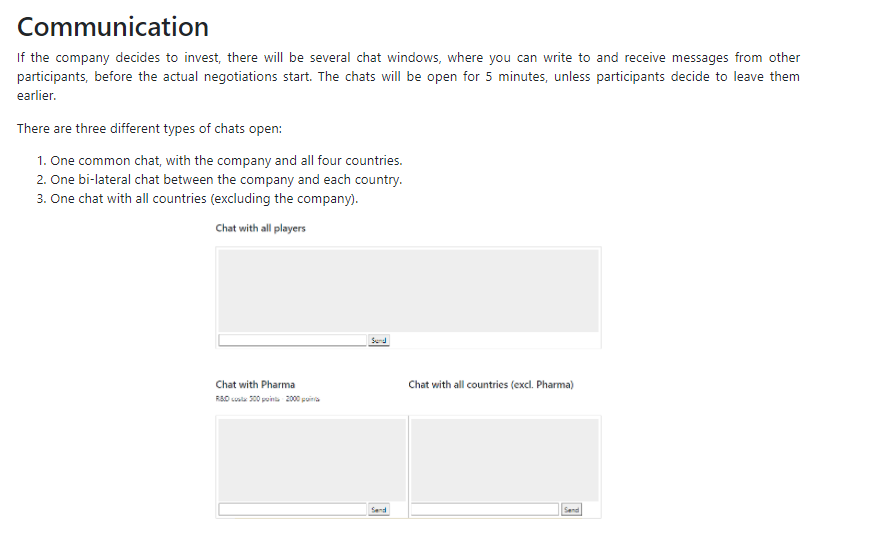


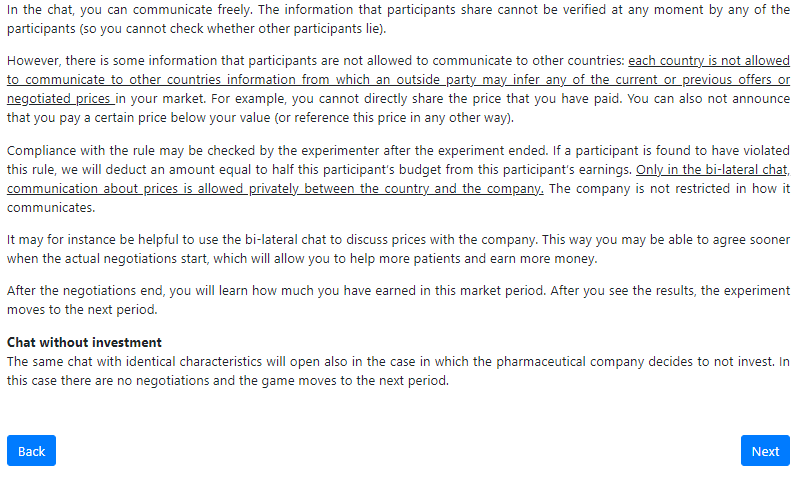
**Page 7 – experimental arm 2 “Price transparency” – NL**


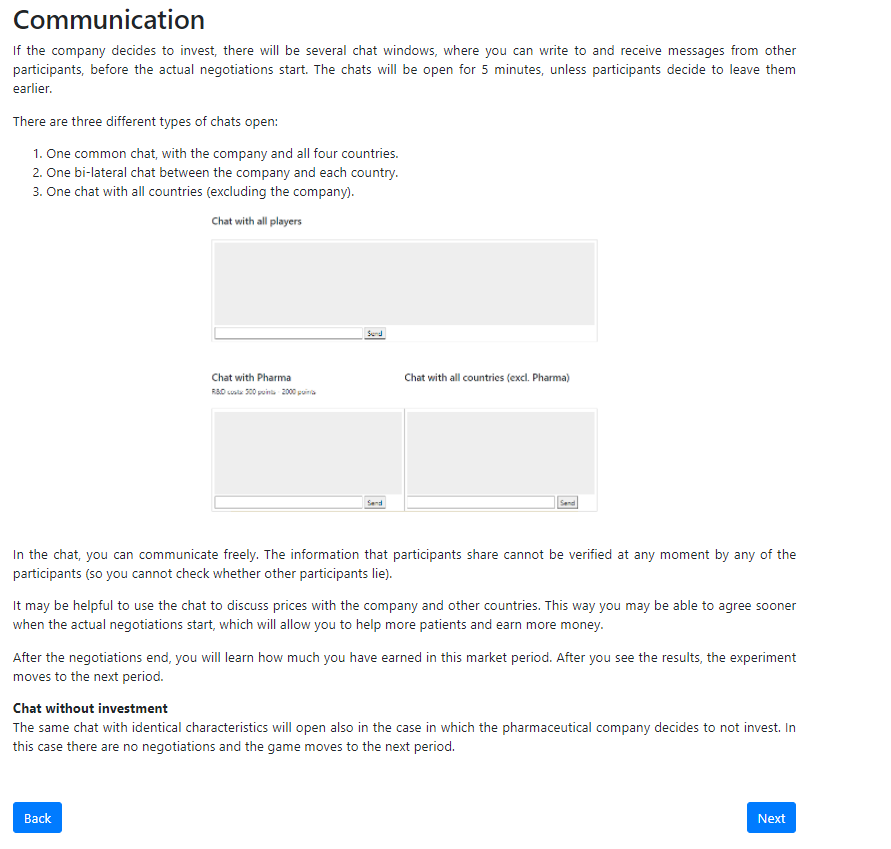


**Page 7 – experimental arm 3 “Full transparency” – NL**


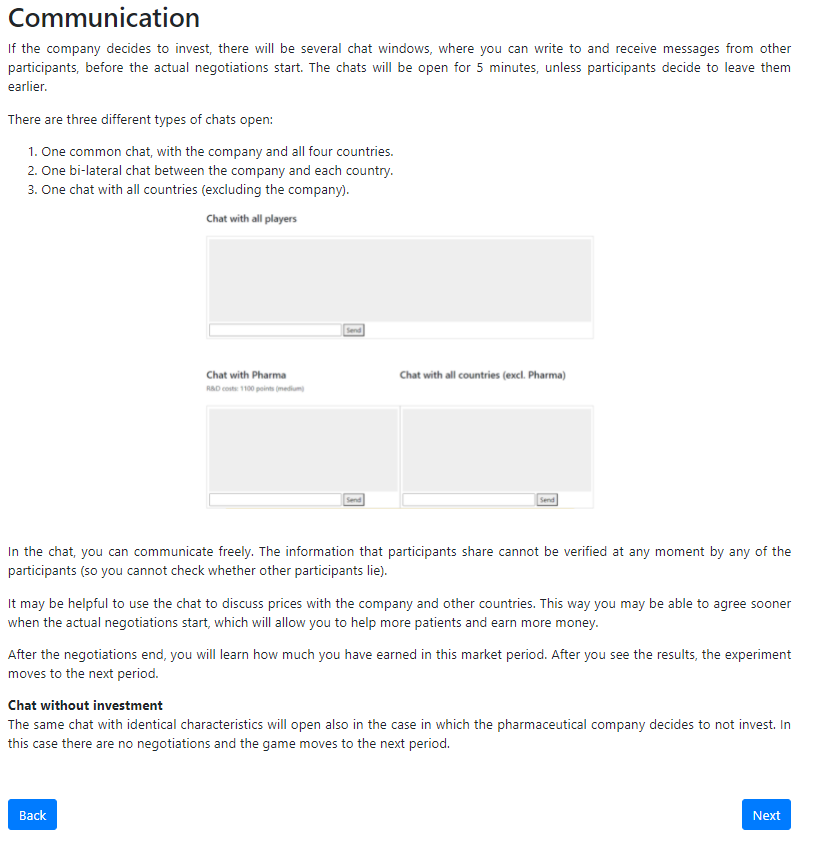


**Quiz – all experimental arms* - Pharmaceutical company**

*Note: only the answer to question 5 differed between experimental arms


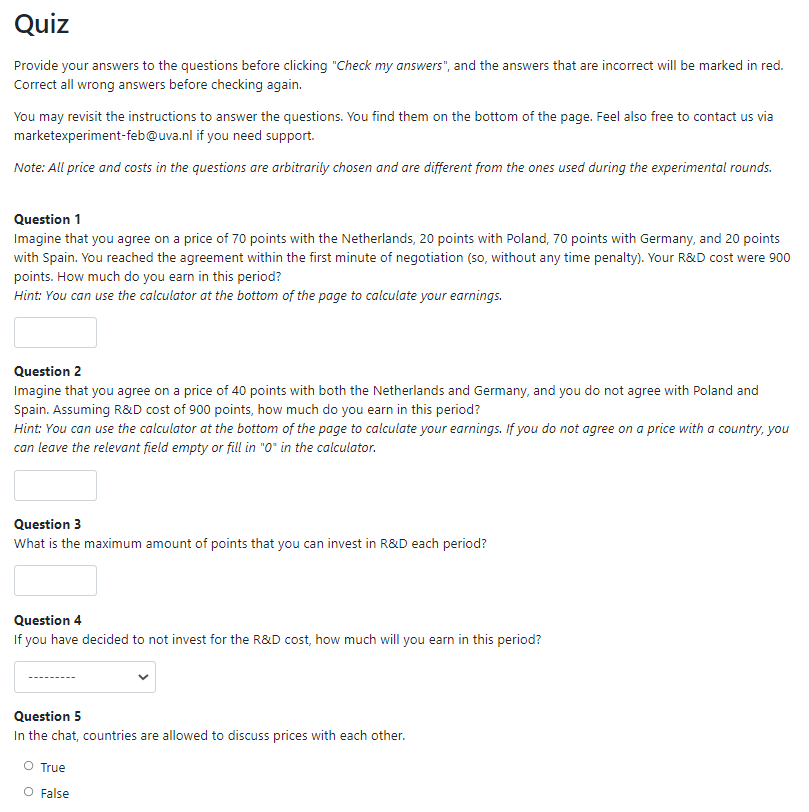


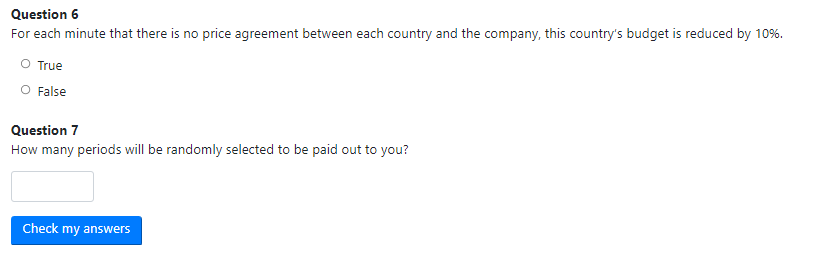

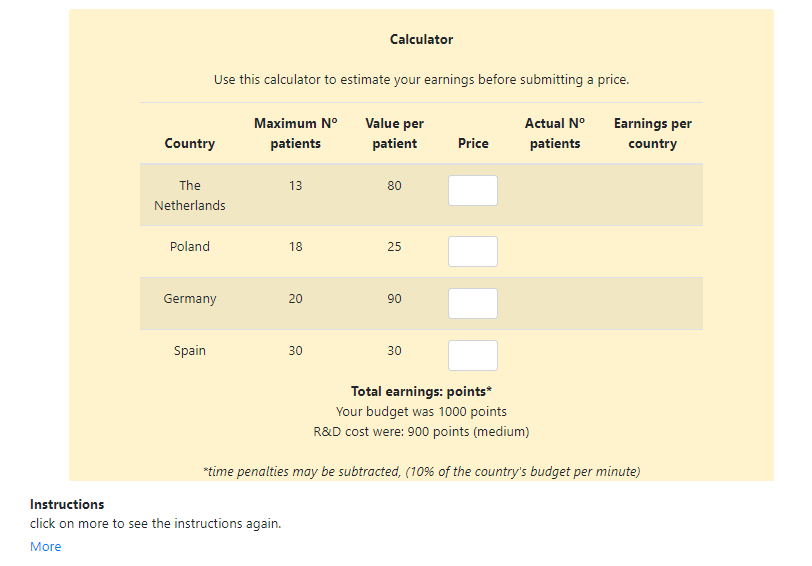


**Quiz – all experimental arms* - NL**

*Note: only the answer to question 5 and the display of R&D costs in the calculator that countries could use differed between experimental arms
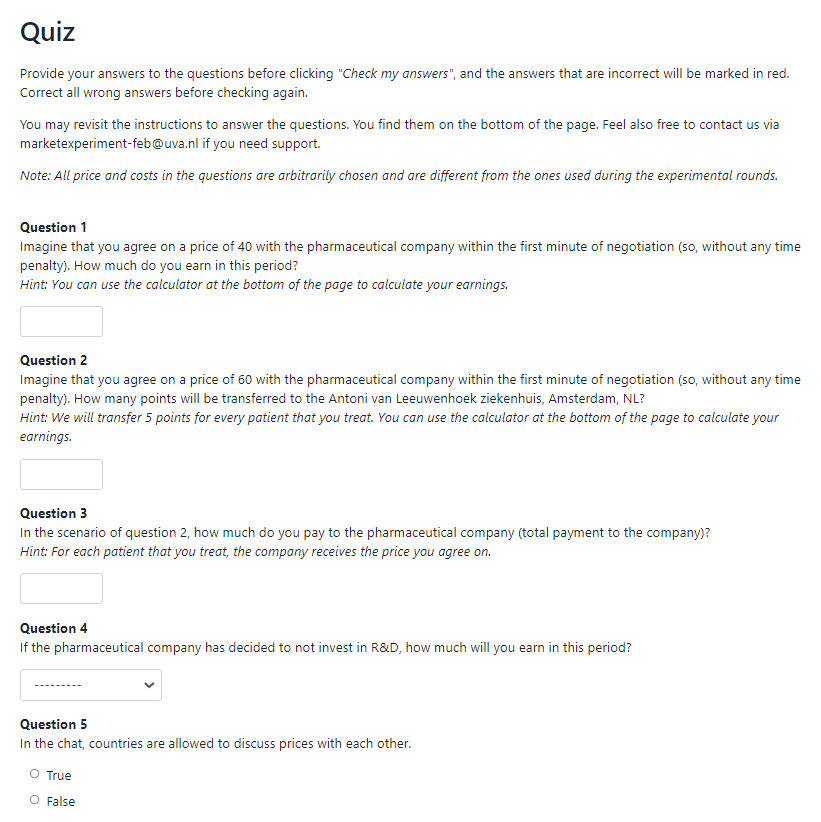

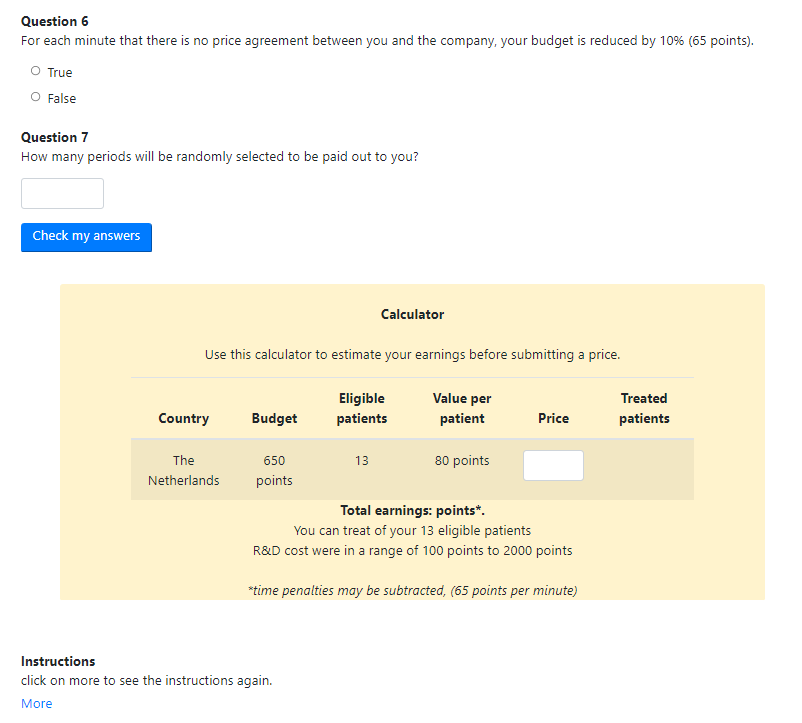

Supplement: Supplementary Data File 2 — Experimental instructions. [file crc-21-0031-s03.docx]
